# Supplementary material for: VAV2 signaling promotes regenerative proliferation in both cutaneous and head and neck squamous cell carcinoma
Source: Nat Commun. 2020 Sep 22;11:4788. doi: 10.1038/s41467-020-18524-3 (PMC7508832; doi:10.1038/s41467-020-18524-3)
Supplement: Supplementary file 1 — Supplementary Information [file 41467_2020_18524_MOESM1_ESM.pdf]

Supplementary Information for

VAV2 SIGNALING PROMOTES REGENERATIVE PROLIFERATION IN BOTH CUTANEOUS AND HEAD AND NECK SQUAMOUS CELL CARCINOMA

by

Lorenzo-Martín *et al*.

This PDF file includes:

Supplementary Figures 1 to 9 and legends (pages 2 to 15)

Supplementary Tables 1 to 5 (page 16 to 26)

Supplementary Note 1 (page 27)

Supplementary References (page 28)

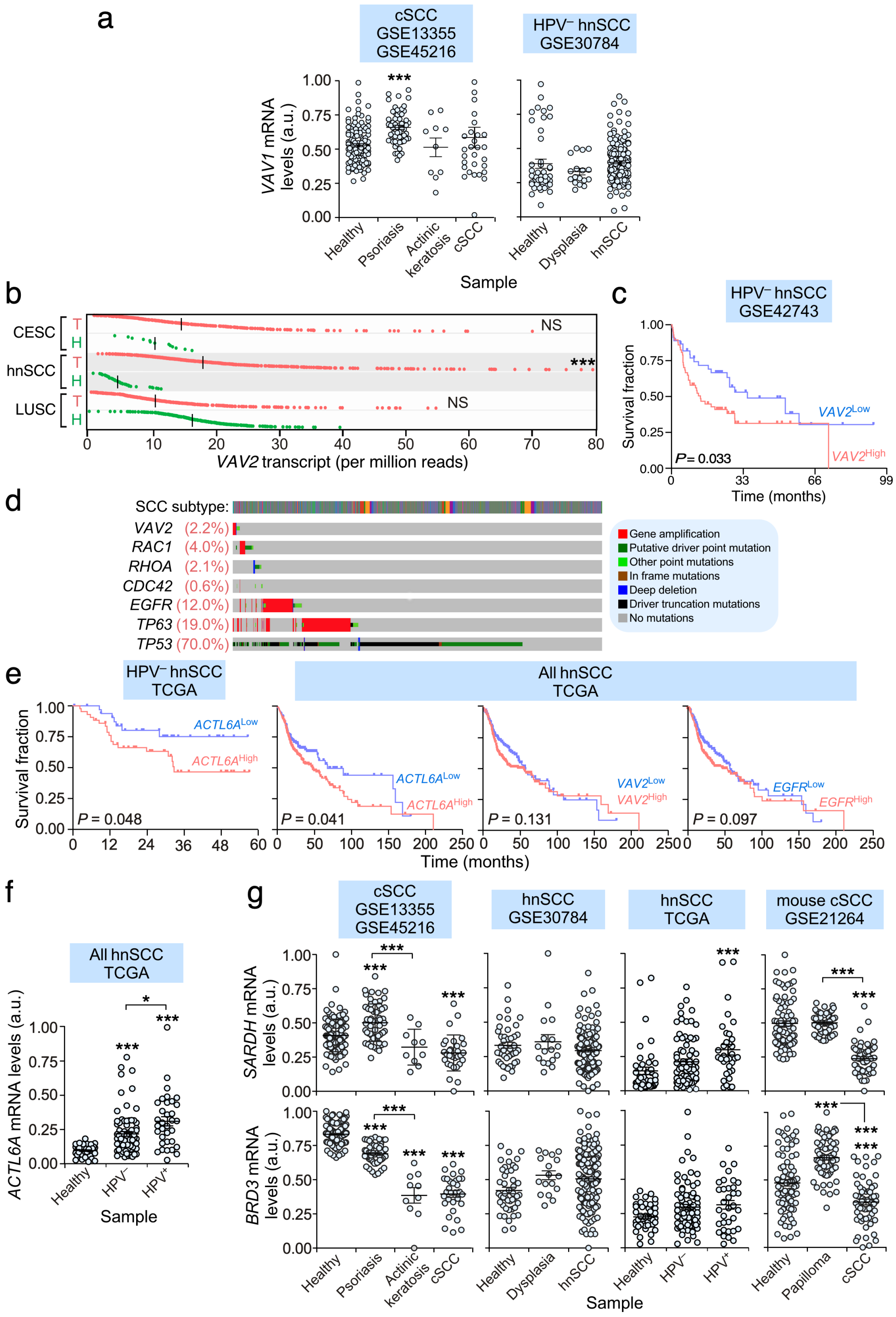

SUPPLEMENTARY FIGURE 1. Increased abundance of VAV2 is associated with poor prognosis in SCC

(a) Dot plot showing the normalized expression of the *VAV1* mRNA in indicated hnSCC subtype patients (bottom). The gene expression dataset used for these experiments is shown at the top. ***, $P < 0.0001$ (ANOVA and Tukey's HSD tests, n = 220 and 229 patient samples, respectively).

(b) Dot plot showing the *VAV2* mRNA abundance in healthy (n = 44) and tumor samples (n = 519) from the indicated cervical (CESC), head and neck, and lung (LUSC) squamous cell carcinoma TCGA datasets (left). ***, $P < 0.0001$. NS, not statistically significant (Student's t-test).

(c) Survival of the hnSCC patients from the indicated datasets according to the abundance of the *VAV2* mRNA present in tumors. The Mantel-Cox test P value is indicated.

(d) Genetic alterations found in the indicated genes (left) in human cSCC and hnSCC patients present in the cBioPortal database. The percentage of samples with gene alterations (in %) are indicated on the left in red.

(e) Survival of the hnSCC patients from indicated datasets according to the abundance of *ACTL6A*, *VAV2* and *EGFR* mRNAs in tumors. The Mantel-Cox test P value is indicated for each transcript.

(f) Dot plot showing the normalized expression of the *ACTL6A* transcript in indicated hnSCC subtype patients (bottom) according to HPV status. The gene expression dataset used for these experiments is shown at the top *, $P = 0.010$; ***, $P < 0.0001$ (ANOVA and Tukey's HSD tests, n = 155 patient samples).

(g) Dot plots showing the normalized expression of the *SARDH* and *BRD3* mRNAs in indicated tissue samples (bottom) and gene expression datasets (top). ***, $P < 0.0001$ (ANOVA and Tukey's HSD tests, n = 220, 229, 155 and 273 patient samples, respectively).

In a, f and g, data represent the mean ± SEM. Source data for this figure are provided as a Source Data file.

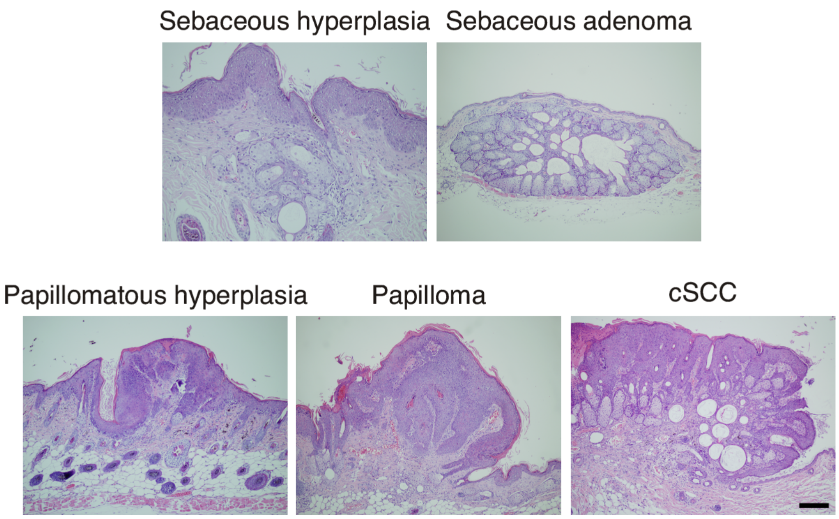

SUPPLEMENTARY FIGURE 2. Examples of the type of tumors detected in WT and *Vav2*^{Onc} mice that have been used to generate the graphs shown in **Figure 3g**. *n* = 87 independent stainings. Scale bar, 200 µm.

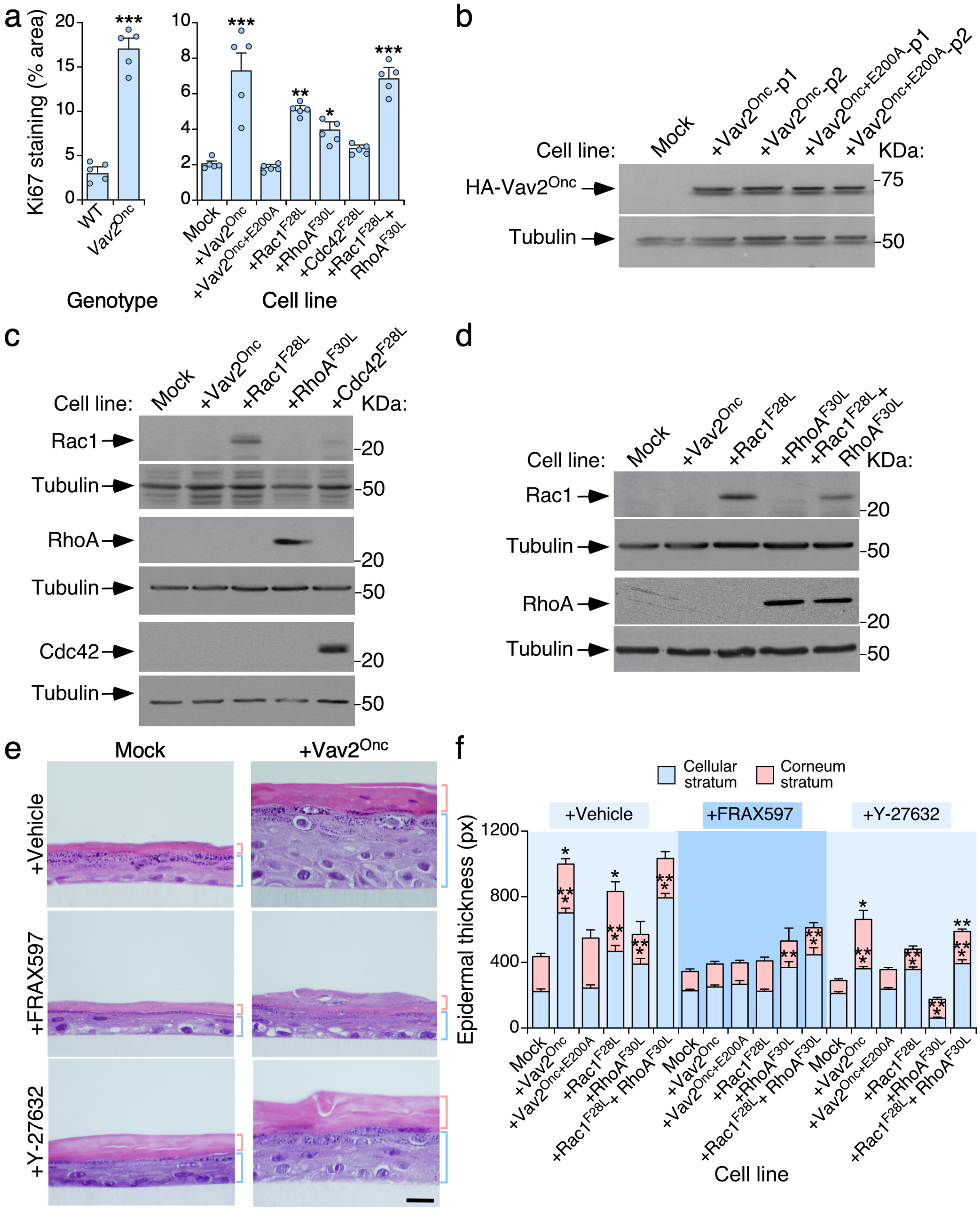

SUPPLEMENTARY FIGURE 3 The effect of Vav2^{Onc} in epidermis is both keratinocyte autonomous and catalysis-dependent

(a) Quantification of the Ki67 immunoreactivity obtained in **Figures 4a** (left) and **4c** (right). *, $P = 0.012$; **, $P = 0.001$; ***, $P < 0.0001$ (ANOVA and Dunnett's multiple comparison test, $n = 5$ independent cultures).

(b, c and d) Immunoblots showing the expression of the indicated proteins in human keratinocytes. Tubulin was used as loading control in all cases ($n = 3$ independent experiments). In B, p1and p2 refer to two independent pools of cells.

(e) Representative images of hematoxylin-eosin-stained organotypic cultures generated by the indicated cells (top) and under the specified experimental conditions (left). The thickness of the corneum and cellular

epidermal strata is indicated with red and blue brackets, respectively. Scale bar, 10 μm (*n* = 3 independent cultures).

(f) Quantification of the thickness of the indicated skin strata according to the data obtained in panel e. px, pixel. *, *P* = 0.041 ($Vav2^{Onc}$, vehicle, corneum stratum), 0.025 ($Rac1^{F28L}$, vehicle, corneum stratum), 0.048 ($Vav2^{Onc}$, Y-27632, corneum stratum); **, *P* = 0.002 ($RhoA^{F30L}$, FRAX597, cellular stratum), 0.009 ($Rac1^{F28L}$+$RhoA^{F30L}$, FRAX597, corneum stratum); ***, $P < 0.0001$ (all other tests) (ANOVA and Dunnett's multiple comparison test, *n* = 3 independent cultures). In a and f, data are given as the mean ± SEM.

Source data for this figure are provided as a Source Data file.

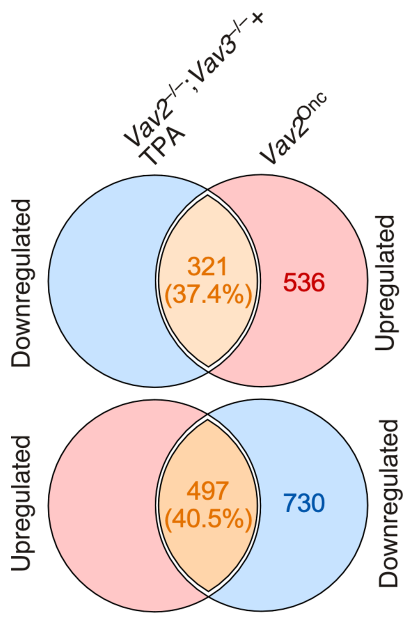

SUPPLEMENTARY FIGURE 4. Vav2 controls a protumorigenic stem cell-like program in keratinocytes

Venn diagram comparing the *Vav2*^{Onc}-regulated transcriptome and the Vav2;Vav3-dependent fraction of the TPA-stimulated gene expression program in the skin of mice. The number of probesets and level of overlap are indicated.

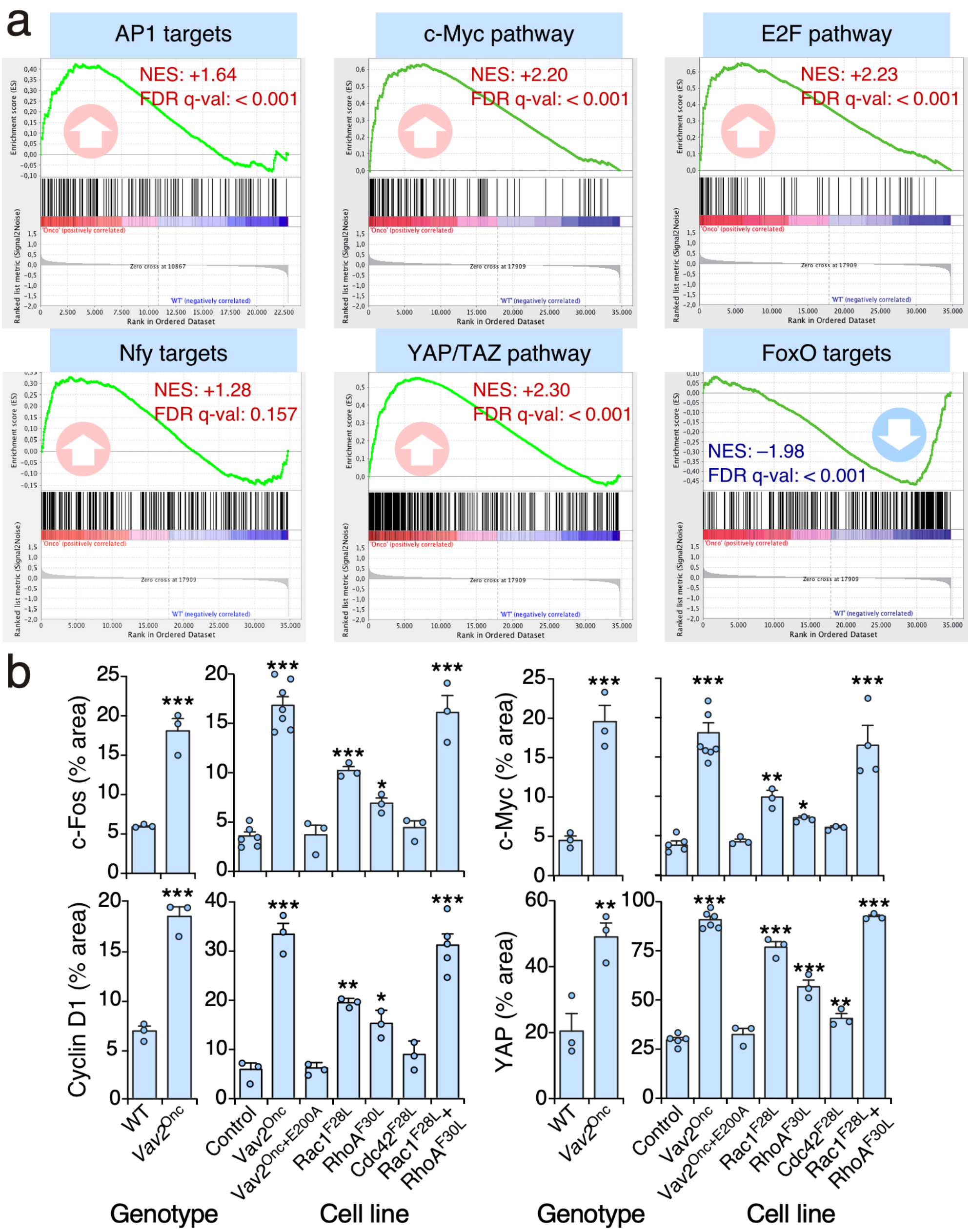

SUPPLEMENTARY FIGURE 5. The *Vav2*^{Onc}-dependent transcriptome is regulated by c-Myc, the YAP/WWTR1/TEAD complex, E2F, and AP1 factors

(a) GSEA of the indicated transcriptional factor-associated gene targets in the *Vav2*^{Onc}-dependent transcriptome. The NES (normalized enrichment score) and FDR (false discovery rate) values are indicated within each graph (GSEA statistical test). Positive and negative enrichments are indicated with upward- and downward-pointing arrows, respectively.

(b) Quantification of the immunoreactivity for the indicate proteins obtained in the experiments shown in **Figure 6c,d**. Data are given as the mean ± SEM. *, $P = 0.017$ (RhoA^{F30L} and c-Fos), 0.011 (RhoA^{F30L} and c-Myc), 0.041 (RhoA^{F30L} and Cyclin D1); **, $P = 0.003$ (Rac1^{F28L} and c-Myc), 0.002 (Rac1^{F28L} and Cyclin D1), 0.009 (Cdc42^{F28L} and YAP); ***, $P < 0.0001$ (all other tests) (ANOVA and Dunnett's multiple comparison tests). $n = 3$ independent cultures.

Source data for this figure are provided as a Source Data file.

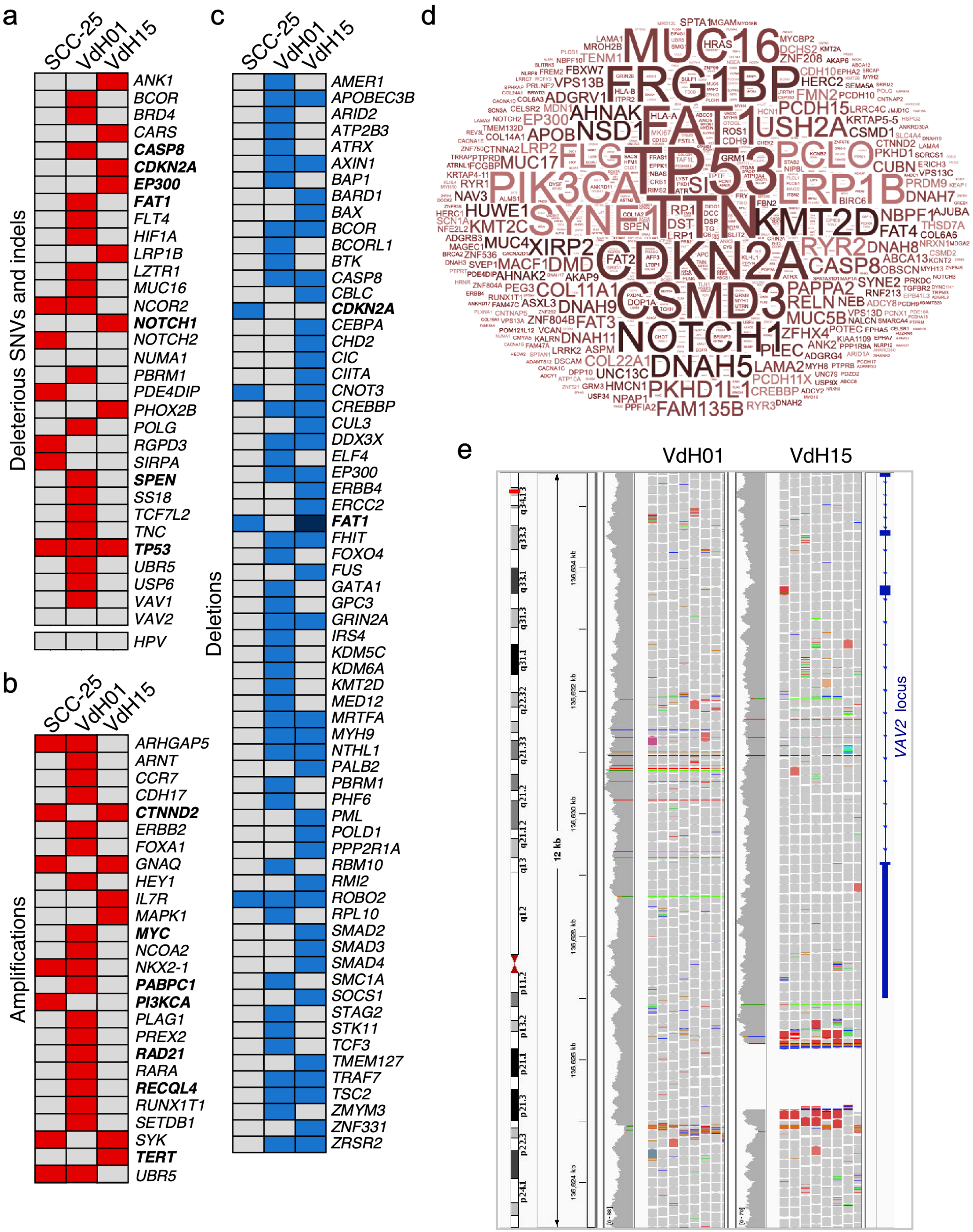

SUPPLEMENTARY FIGURE 6. Characterization of the main genetic alterations found in VdH01 and VdH15 patient-derived cells.

(a to c) Heatmaps showing the deleterious single nucleotide variants (SNVs) and indels (a, red boxes), amplifications (b, red boxes), and deletions (c, blue boxes) identified in the genome of the indicated cells (top). The data for the SCC-25 cell line have obtained from the Ref. #36 cited in the main text (Martín *et al.*, 2014). The genomic lesions that occur in at least 5% of hnSCC patients according to the TCGA dataset are highlighted in bold. In all cases, absence of mutations is depicted as a gray box.

(d) Wordcloud representing the most frequently mutated genes in hnSCC patient samples according to the TCGA data. The size of each gene symbol is proportional to the mutation frequency of that gene in hnSCC.

(e) Genomic view of the 3' genomic regions that is downstream of the *VAV2* gene in the indicated hnSCC patient-derived cell lines (top). From left to right, we show the chromosome map, the sequencing coverage, and the alignment of reads.

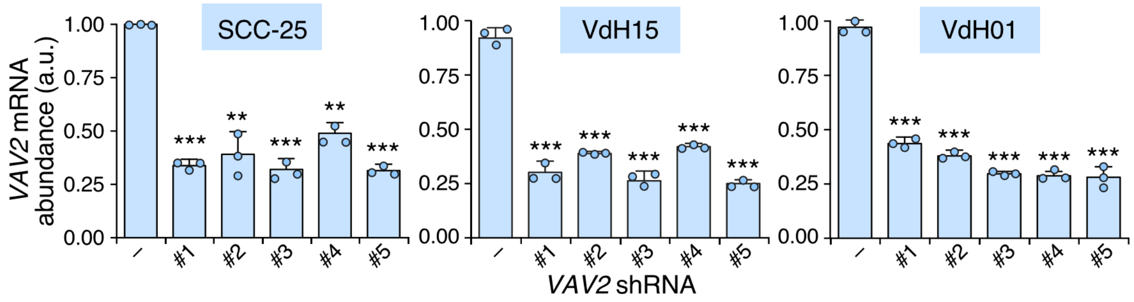

SUPPLEMENTARY FIGURE 7. VAV2 downmodulation in human SCC patient-derived cells. Level of *VAV2* mRNA knockdown achieved by the indicated *VAV2* shRNAs (bottom) in SCC-25, VdH15, and VdH01 cells. **, $P = 0.009$ (SCC-25 #2), 0.007 (SCC-25 #4); ***, $P < 0.0001$ (all other tests) (ANOVA and Dunnett's multiple comparison test, $n = 3$ independent experiments). Data are given as the mean ± SEM.

Source data for this figure are provided as a Source Data file.

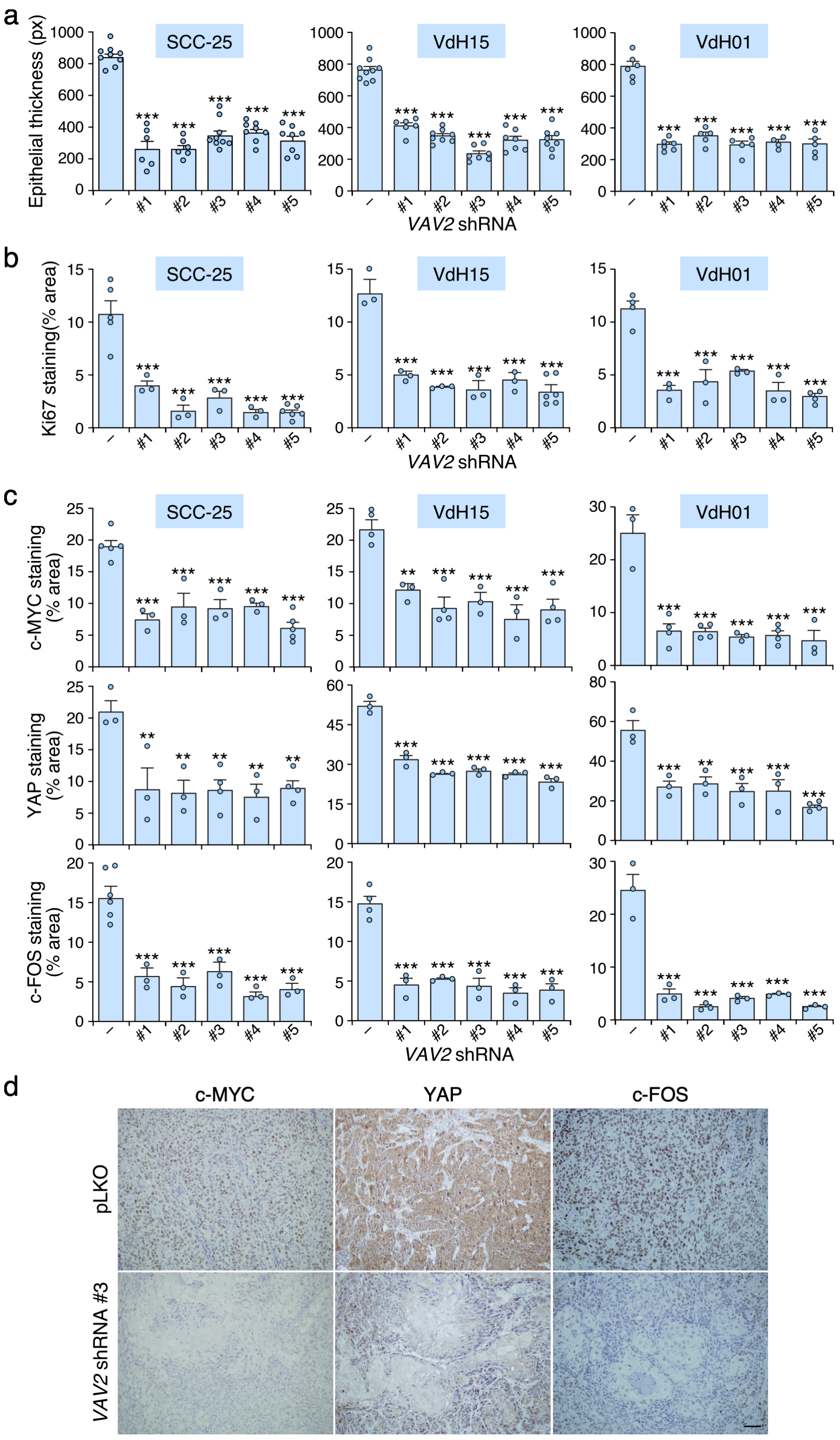

SUPPLEMENTARY FIGURE 8. VAV2 downmodulation abrogates the tumorigenic potential of hnSCC patient-derived cells

(a and **b)** Quantification of epithelial thickness (a) and Ki67 immunoreactivity (b) in the organotypic cultures displayed in **Figure 9a**. ***, $P < 0.0001$ (two-sided Student's *t*-test). $n = 5$ (a) and 3 (b) independent cultures. Data represent the mean ± SEM.

(c) Quantification of the immunoreactivity for the indicated proteins (left) in the specified control and *VAV2* shRNA-knocked down cells (top). The *VAV2* shRNA used (#1 to #5) is indicated at the bottom. **, $P = 0.003$ (VdH15 #1, c-MYC), 0.009 (SCC-25 #1, YAP), 0.007 (SCC-25 #2, YAP), 0.001 (SCC-25 #3, YAP), 0.005 (SCC-25 #4, YAP), 0.001 (SCC-25 #5, YAP), 0.009 (VdH01 #2, YAP); ***, $P < 0.0001$ (all other tests) (ANOVA and Dunnett's multiple comparison tests, $n = 3$ independent cultures). In all panels, data are given as the mean ± SEM.

(d) Lower magnification of the tumor sections shown in **Figure 9g**. Scale bar, 50 µm ($n = 8$).

Source data for this figure are provided as a Source Data file.

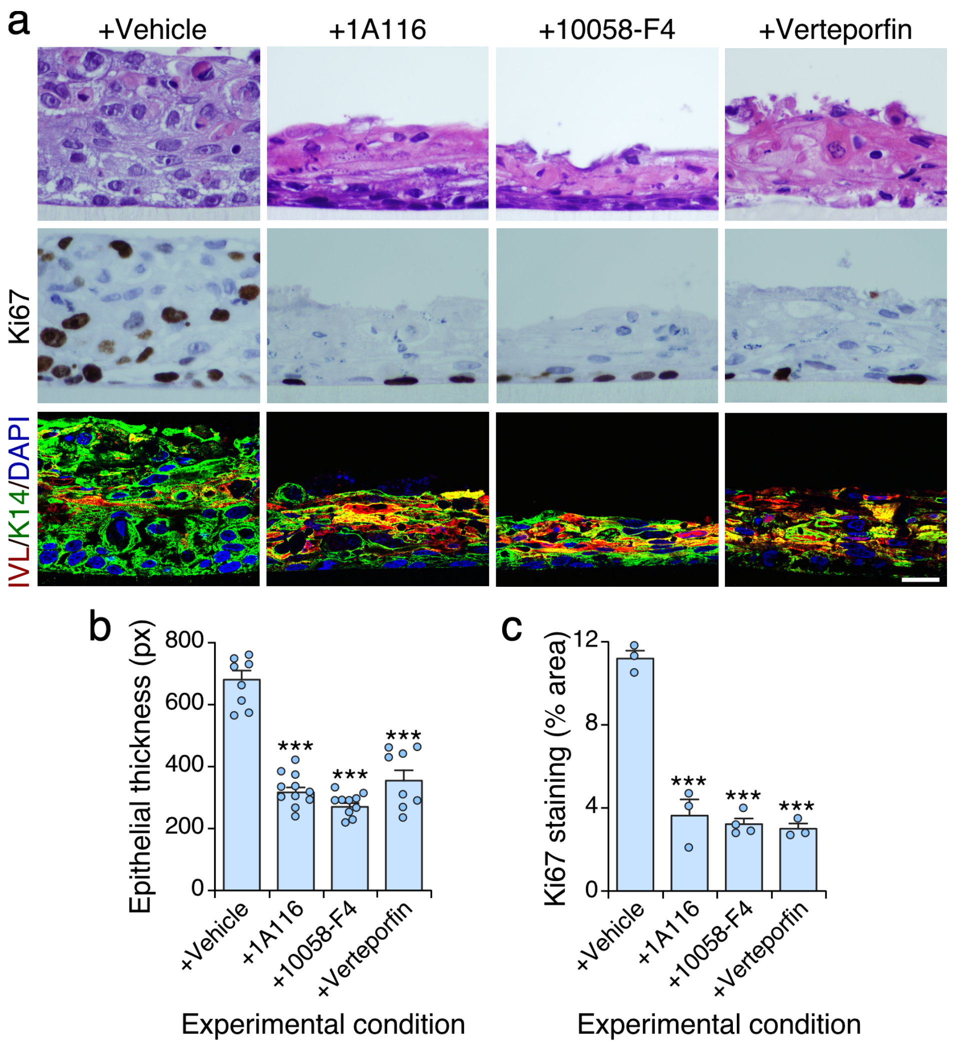

SUPPLEMENTARY FIGURE 9. The impact of the depletion of endogenous VAV2 in oral SCC PDCs is mimicked by the chemical inhibition of endogenous RAC1, c-MYC and YAP/TEAD complexes

(a) Representative images of organotypic cultures of parental VdH15 cells subjected to the indicated experimental conditions (top) upon staining with either hematoxylin-eosin (top panels) or antibodies to Ki67 (middle row of panels), IVL (bottom panels, red color) or K14 (bottom panels, green color). Some sections were counterstained with either hematoxylin (middle row of panels) or DAPI (bottom panels, blue color). Scale bar, 10 µm (n = 3 independent cultures).

(b and **c)** Quantification the epithelial thickness (b) and Ki67 immunoreactivity (c) obtained in the experiments shown in panel a. ***, P < 0.0001 (ANOVA and Dunnett's multiple comparison test, n = 3 independent cultures). In b and c, data are given as the mean ± SEM.

Source data for this figure are provided as a Source Data file.

SUPPLEMENTARY TABLE 1. Transcriptomic datasets used in this study

ACCESSION NUMBER	REFERENCE	SPECIES	SITE	SAMPLES	HEALTHY TISSUE	HPV STATUS	SURVIVAL INFO
GSE21264	PMID: 21244661	Mouse	Skin	273	Yes	Not applicable	No
GSE13355 & GSE45216	PMID: 19169254 & PMID: 24335922	Human	Skin	220	Yes	Not reported	No
GSE30784	PMID: 18669583	Human	Head and neck	229	Yes	Not reported	No
GSE41613	PMID: 23319825	Human	Head and neck	97	No	Negative	Yes
GSE42743	PMID: 23319825	Human	Head and neck	103	Yes	Negative	Yes
TCGA HNSCC	PMID: 25631445	Human	Head and neck	563	Yes	Positive & Negative	Yes
TCGA LUSC	PMID: 22960745	Human	Lung	824	Yes	Not applicable	Yes
TCGA CESC	PMID: 29625050	Human	Cervix	319	Yes	Not applicable	Yes
GSE52651	PMID: 25805135	Human	Epidermis	21	NA	Not applicable	Not applicable
GSE26713	PMID: 21481790	Human	T–ALL	124	Yes	Not applicable	No
GSE30219	PMID: 23698379	Human	Lung	307	Yes	Not applicable	Yes

SUPPLEMENTARY TABLE 2. Genes belonging to the Vav2^{Onc} redundant and nonredundant transcriptomes relative to the Vav2+Vav3-dependent fraction of the TPA-stimulated gene program in the mouse skin

**REDUNDANT (Up in Vav2^{Onc})**
Cenpn, NA, Gas5, Pdap1, Pitrm1, Cdc20, Fam162a, Kpna2, Hmgb3, Cct3, Tmem97, Hist1h4c, Itgb4, Hist1h2bh, Prmt1, Rpl38, Urah, Gnl3, Mcm3, Nop56, Hmgb2, Ftsj3, Ahsa1, Ddx21, Lbr, Mybl2, Dsg3, NA, Mthfd1l, Hist1h2be, Cct5, Ppm1g, Hist1h4m, Snrpf, Nt5dc2, Hmgb1, Rps8, Slc7a5, Tk1, Cenpm, Eef1e1, Srm, Rrm2, Nup93, Snord16a, NA, Aurkb, Fbl, Noc2l, Alyref, Rcc1, 2610528A11Rik, Myo10, Stom, Pak1ip1, Trip13, Erh, Nek2, Hmgb2, Gm3893, Mcm10, Snrpg, Myc, Rpl38, Hmgb1, Srsf9, Gm2173, Gm3696, Hspe1, Snrpd1, Hmgb1, Ccne1, Hmgb1, Nudc, Aurka, Hmgb1, Pla2g4c, Cdca5, Hnrnpa3, 4933409K07Rik, Ppih, Tmem97, Cks2, Rps12, Rplp1, Ndc80, Mki67ip, Myl4, Hist1h2ao, Rpl24, Cdk1, Snora62, Ranbp1, Cdca8, Wdr12, Ldlr, Mlana, Mybbp1a, Hmgb1, Hist1h2br, Myh9, Hist1h4h, Enah, Kif22, Col18a1, Rad51ap1, Sprr1b, Hyal1, Ldlrap1, Cad, Cks1b, Birc5, 9430008C03Rik, Rnu12, Snora74a, H2afx, Nup43, Orc1, Rcl1, Kpna2, Itgb1, Tars, Incenp, NA, Hmgb2, Exosc2, Duox1, Impdh2, Heatr1, Serpinb6c, Gas5, Ass1, Ube2s, Mcoln3, Hist1h2ab, Rpl12, Wfdc12, Hmgn2, Hist1h4b, 9430008C03Rik, Hmgb1, Hmgb1, Ppan, Nop16, Bnc1, Grwd1, Has3, Bcl2l15, Nrp2, Larp4, Snora33, Itga3, Nolc1, 4933409K07Rik, Pmel, Mt1, Scd2, Lrp8, Itpa, Rnu3b1, Myh9, Hirip3, Slc24a5, Ppil1, Slc45a2, Lmnb1, Gm3002, Ccnd1, Snora61, Pola1, Gm5416, Cth, Slc20a1, Snora65, Ube2s, Ctps, Fhl2, Col12a1, Timm8a1, Adamts1, E2f7, Ptprz1, S100a6, Serpina9, Hnrnpa3, Pi15, Npm3, Timm8a1, Vaultrc5, Fscn1, Idi1, Erh, Lcn2, Dusp6, Mthfd2, Odc1, Hist2h2ab, Mrto4, Hmgb1, Snord53, Tnfrsf12a, Ccdc86, Junb, Tslp, NA, Fen1, Gjb6, Etv4, Krt35, Bcl3, Tubb6, Gsta4, Bcat1, Gjb2, S100a8, S100a9, Odc1, Lipg, Gsr, Hbegf, Hp, Akr1b8, Il33, Ahcy, Dct, Cxcr2, Snord15a, Tyr, Ngf, Rps27a, Ccnb1ip1, Snord49a, Krt85, Itga5, Socs3, Mrto4, Smim3, Procr, Snora31, Tnc, Slc7a11, Rnu73b, Wif1, Fosl1, Gm5483, Rnu3a, 4933409K07Rik, 5730408K05Rik, Hist1h3g, Clcf1, Fbp1, Defb14, Phlda1, Lamc2, Krt28, Sh2d5, Tubb3, Tyrp1, Saa3, Cdhr1, Cntfr, Pdpn, Saa2, Ms4a4c, Lrat, Areg, 4933409K07Rik, Mmp9, Sirpb1b, Rnu1b1, Nrg1, Slc39a14, Slpi, Krt73, Inhba, Plek, Gm5150, Serpine1, Fam26d, Ifitm1, Timp1, Ptgs2, Trem3, Mmp13, Slc2a3, Ifitm1, Krt31, Epgn, Cyp4f18, Osm, Ifi202b, Krt33a, S100a3, NA, Krtap11-1, Mmp1b, Plaur, Hdc, Krt27, Krtap19-5, Krt71, Upp1, Ccl4, Krt25, Slfn4, Il1b, Prg4, Krtap19-1, Cd14, Spatc1, Nlrp3, Ccl3, Selp, Spp1, Trem1, Krtap15, Clec4d, Il24, Irg1, Cxcl2, Clec4e, Il6
**REDUNDANT (Down in Vav2^{Onc})**
Skint9, Skint10, H2-T22, Krt77, Ankrd35, Inmt, Skint11, H2-Q6, Cyp2g1, Sema3d, Clca1, Serpina3b, Alox12e, Skint4, Il22ra2, Serpinb7, Bbox1, Trdn, Il20ra, Clca2, Agbl1, Ucp3, Abca5, Cacna1s, Xirp2, Tcrg-V1, Pkia, Irf4, Scd3, NA, Mettl21c, Mlf1, Mstn, Mmp27, Oas1f, Trgv2, Uprt, H2-Ab1, Myh2, Ampd1, Scn2a1, Myom1, Asb12, Ccr4, Skint7, Flg2, Wnt3, Slc2a12, Ecm2, Skint2, Sbk2, Calm5, Klhl31, Myo18b, Lipn, Ereg, Tmod4, Kcnj2, Myoz1, Filip1l, Scn2a1, 2300005B03Rik, Smyd1, Mrgprb2, Clca3, Chit1, Mybpc1, Lce1m, Fbxo32, Skint3, Cmbl, 9430007A20Rik, Ccdc3, Txlnb, Cyp2b19, Xkrx, Fsd2, Slc5a9, Pdk4, Krt78, Serpinb12, Tmem182, Aloxe3, H19, Casq1, Il20rb, Slurp1, Dab1, Sgcd, Lce1a2, Zfp943, Ttn, Trim7, Lce6a, Myot, H2-Eb1, Serpina12, Ppp2r3a, Trdn, Klhl38, H2-M2, Clca5, Tmem159, Otop3, Slc15a2, Mill1, Lrrc39, Satb1, Ckmt2, Neb, Ikzf2, Gal3st4, P2ry4, Tgfb2, Cd55, Slc16a14, Adh6a, Exph5, Srl, Lce1l, 9530053A07Rik, Sypl2, Lce1i, Osr2, Myom3, Cttnbp2, Pcmtd1, Ear2, Lce1f, Myh1, A930018M24Rik, Ogn, Slc24a3, Pdk2, Lor, Gdpd2, Lce1d, Pygm, C4b, Sh3rf2, Klhl31, Atp1b1, Lrrc28, Il20rb, Lmod2, Hal, Fndc1, Lipk, Cpxm2, Them5, Adck3, Fmo2, Acvr1c, H2-Aa, Sox6, Rb1cc1, B3galt2, Il12rb2, Zbtb16, Cilp, Pou2f3, Mfap4, Lipm, Cobl, Map2, Lce1g, Fam214a, Mettl7a1, Nr1d1, Plekhn1, Thrb, Lmod3, Gm7165, Krt15, Ccl27a, 2310014L17Rik, Slc28a3, Klhl24, Fndc9, Gm11992, Gpld1, Il1f8, Fitm1, Il20rb, Krt10, Aif1l, Rps6ka6, Mrgprb3, Smpd3, Nipal2, Fam189a2, Slitrk6, Il1f6, Gm7609, Tmem229b, Tstd2, Atp1a2, Tmprss11f, Calcb, Nt5c3, Cyp27a1, Ear5, Plb1, Kcnj16, Acot2, Myom2, C6, Cyp2f2, Rora, Gm5105, Nrap, Serpinb11, Ar, Gpr111, Sh3gl2, 2310007B03Rik, Cyp2r1,

Tjp3, A030009H04Rik, Atp10b, Lrrc61, Gramd1c, Slc6a4, Fam3b, Scara5, Eps8l3, Slco2b1, Pck1, H2-DMa, Lce1c, Cyp2j9, Eps8l1, Alox12, Maob, Actn2, Rasgrp1, Aldh1a7, Clec3b, Hyal4, ago-04, Dlc1, Ccl19, Ano9, Retnla, Cpt1b, Krt23, Calcoco1, Papl, Trp53inp1, Dapp1, Slc34a2, Fam134b, Kctd4, Hhatl, Serpinb8, Mettl7a3, Il1f5, Acpp, Orm1, Dapl1, NA, Myoz2, Kprp, Opcml, Aldh1a1, Nkg7, Cyp2j6, Tmem229b, Asah2, Nccrp1, Pkib, Cdo1, Rnf167, Slc22a4, Myo6, Gata6, Gyg, Hmgcs2, Atp6v1c2, Hspb6, Calm4, Gpcpd1, Yod1, Mboat2, Olah, Frmpd1, Gba2, Adh1, Tprg, Ggct, Apobec2, Asb2, Ssbp2, Pcolce2, Acss1, Anxa9, Il18, Arhgap40, Sgpp1, Liph, Ahnak2, Sema3c, Ptgs1, Kdm6a, Pnpla3, Cpa4, Esyt3, Slco3a1, Nlrp10, Gsdma2, Cpm, Scgb1b27, Pcmtd2, Arhgef37, Coro2b, Arhgap20, Arrdc3, Slc36a2, Leprel1, Gprin2, Tppp, H19, Rnase1, Cox6a2, Fxyd1, Fmo5, Myo18b, Unc93a, Adamts14, Dkkl1, Cd109, Car14, Casp14, Gpc6, Scel, Abca6, Mup3, Acsbg1, H2-DMb2, Mpz, Mfap2, Fyco1, Lce1b, Cd3e, Slc46a2, Faah, Dnase1l2, Slc16a9, Esr1, Hbp1, Sdcbp2, Glcci1, Slc25a12, Elmod1, Chga, Hsd17b14, Gzmc, Susd2, Ciita, Dlg2, Ezh1, Cgn, Gpr115, Plekha5, Oxtr, Atg13, Foxn3, Klk11, Gprc5c, Trp53inp2, Ccl17, Pof1b, Plcxd2, Gan, Ldb3, Itm2a, 2310042D19Rik, Adssl1, A530099J19Rik, Ttc28, Hrnr, A530016L24Rik, Agpat5, Zfand6, Ascl2, Cat, Camsap3, Acer2, Abca9, S1pr5, Ak1, Inpp5k, Ptpn14, Tppp3, Fcho1, Ypel2, Aff3, Cd74, Bmp2, Gpd1, Lrrn4cl, Dyrk1b, Lnx1, Arhgef5, Ivl, Galnt15, Plxdc2, Abcc5, Nuak1, Hoxa9, Lce1a1, Nlrc5, Slc31a2, Rgmb, Slc43a1, Ephx3, Slc15a1, Gmpr, Pink1, Card14, Slc37a2, Nnt, Cdon, Tob1, Plb1, Tcea3, Hiatl1, Lce1h, Sft2d2, Acsl1, Slc27a1, Akr1c14, Gsdma, Abca12, Pdzk1ip1, Gjb5, Nlrc5, Mr1, Crebrf, Camk1d, Cited2, Npy1r, Rbp4, Ivd, Atp12a, Gdpd3, Klf9, Nqo2, Alox12b, Nrd1, Plb1, Ust, Txnip, Ctnnbip1, Dhrs3, Mfap3l, Slc22a5, Adam23, Nebl, Tnxb, Sgpl1, Prss8, Acadm, Mup6, Sik1, Pik3cb, Efna3, sept-03, Tspan5, Bpifc, Sntb1, Itfg3, Hmcn1, Mid2, Dexi, Slc46a3, Dpp4, Tmprss4, Atg9b, Reck, Slc9a9, Fut2, Agpat4, Pepd, Cd59a, Ppl, Sdr9c7, Slc26a9, Cul3, Hfe2, Tmem170, Adamtsl1, Myzap, Timp3, Spon2, Dennd1b, Klk9
**NON-REDUNDANT (Up in Vav2^{Onc})**
BC018473, Defb8, Snora20, Actn3, Gas5, BC117090, Myh4, Prss35, Acsm3, Cyp3a57, Uty, Fut11, Echdc1, Tmem56, Krt24, Acoxl, Snhg1, Pank1, Acacb, A130014H13Rik, Ppia, Stfa3, Cenpk, Tbrg3, Pdss1, Glo1, Sdr16c6, Ect2, Hmgb1, Depdc1a, Serpinb3b, Glo1, Igfl3, Atad5, Ptplb, 4933409K07Rik, Hmgb1, Elovl6, Cenpw, Hmgb1, Secisbp2l, Hells, Ddx3y, Hsp90aa1, Rps3a1, Kif23, Acot5, Chek1, Cthrc1, Eif2s3y, Gins1, Npm1, Hmmr, Hspd1, Sc4mol, Hist2h2aa1, Stfa2l1, Hmgb1, Smc4, Dhcr24, Kdm5d, Bub1, Paxbp1, Soat1, Fa2h, 4930422G04Rik, Gm3579, Tlcd2, Rbmxl1, Stil, Taf1d, Wdr76, Idi1, Far2, Slc25a48, Zwilch, Hmgb1, Brca1, Atad2, Myh3, Sgol1, NA, Hist2h3c1, Nucks1, Hnrnpa3, Mad2l1, Elovl3, NA, Ptch1, Rps12, Rs5-8s1, Hmgb1, Casc5, Dtl, Arntl2, Prim1, Diap3, Ncapg2, NA, Kif15, Naa50, Snrpe, Dnaja1, Gen1, Kif14, Supt16, Mki67, Cspp1, Tcp1, Taf1d, NA, NA, Spata5, Anp32b, Rbl1, Chaf1b, Plxnb1, Hmgb1, Rps4x, Gm13178, Cenph, 2210404J11Rik, Snora30, Ncapg, Parpbp, C330027C09Rik, Ccnl1, G2e3, Hspd1, Cdca7l, Rpl38, Ptges3, Ces4a, Kif11, Hmgb1, Ankrd1, Naa25, NA, Mis18bp1, Hmgb1, Esco2, NA, Nusap1, Hist2h2aa1, Kif20b, NA, Pcna, Hnrnpd, Ccnb2, Cyp51, Syncrip, Ppih, NA, NA, NA, Eif2s1, Lss, Srsf1, Hmgb1, Hnrnpu, Rbm25, Hat1, Srsf10, Cenpa, Snora23, Rps25, Srsf7, Dimt1, Rps4x, Hmgcr, Topbp1, Dnajc9, Bora, Cbx3, NA, Awat1, Top2a, Wdhd1, Hmgb1, Npm1, Hist2h2bb, Tk1, Anln, Hsp90aa1, Mcm2, Rps12, Snord35a, Rbm25, Fam60a, Prr11, Racgap1, Pole, Tfap2a, Ticrr, Dgat2l6, Hist2h2aa1, Taf1d, Hmgb1, Ccna2, Sgol2, Hmgb1, Spag5, Kntc1, Xpot, Dlgap5, Snora69, Hmg1l1, Nudt5, 2410002F23Rik, Smc2, Dctd, Kif18a, Fbxo5, Hmgb1, Gyk, Smarcc1, NA, Hnrnpc, 2010107E04Rik, Snord42b, Ttk, Cdca7, Hist1h3g, Hist2h2aa1, Ptch2, Fancd2, Ptma, Tcerg1, 2810417H13Rik, Hist3h2a, Mat2a, BC100530, Suz12, Gyltl1b, Hist1h3d, Cbx3, Hist2h4, Actc1, Mfsd2a, Gm16494, NA, Hist1h2an, Nasp, Gpnmb, Hmgb1, Lap3, Ttll4, Ncapd2, Taf15, Hmgn2, Rps27a, Fabp5, Far1, Dkc1, Psma5, Hnrnpa2b1, Rrm1, Malat1, Snhg5, Dhfr, Mms22l, Rrp1b, Npm1, Pcnp, Rps12, Srsf5, Rpl13, Taf4b, Pold3, Npm1, Itga6, Cd44, Hmgb1, Shmt2, Bdh1, Shcbp1, Eif3a, Ska3, Hspa14, Gm5803, Rps12, NA, Syne2, Slc2a1, Pctp, Hist1h1a, Hmgb1, Snord82, Nup155, Cenpq, Gtf2ird1, Stmn1, Kpna2, Fabp5, Gm5643, Sumo3, Eif4a1, Eno1, NA, Hnrnpa3, Clcn6, Hnrnpa3, Hist2h3b, Kif20a, Blm, Depdc1a, Dnmt1, Krt6b, Hist1h2ao, Ppia, Brca2, Hist1h3a, Arhgap11a, Ptma, Stfa1, 2810417H13Rik, Cbx3, Snord14d, Rps8, Gm5478, Abhd1, NA, Rrs1, Prim2, Rpl24, NA, Mtbp, NA, Taf1d, NA, Prc1, Shfm1, Ccne2, H2afz, Uhrf1, Taf9, Hist1h2ao, Gli1, Ilf2, Plk4, Rps3a1, Nme2, Hnrnpab, Cdkn3, Snhg1, Tpm4, Hist1h3f, Rps4x, Mak16, Tyms, Pa2g4, Snora3, Rangap1, Hmgb1, Cdk2, Cenpe, Hist1h3f, Snord14d, Rps4x, D17H6S56E-5, Rps8, Kif4, Nop58, Rps27a, Anxa2, Bub1b,

Hist1h2ak, Fam60a, Set, Mcm7, Hspa9, Snhg1, Kif2c, Hist1h2br, Rps9, Hist1h2bc, Kcnk1, Mcm5, Hint1, Hist4h4, Tagln2, Gm10052, Hist1h2br, Hist1h4d, Lrrc59, Hnrnpf, Cdc25c, Hist1h4b, Whsc1, Pcna, Mcm6, Lig1, Ube2i, Rad51, Cdca3, Mcts2, Mcm4, Hist1h4f, Syce2, Atp1a1, Nup107, Snrpa1, Shmt1, Rps29, H2afz, Hist1h2af, Dbf4, Ncl, Hist1h2ao, Hist1h3g, Dhx9, Ccnb1, Aqp3, Smpdl3b, Rps3a1, Ppa1, Rps8, Hist1h1b, Tnnt2, Ppih, Rbmxl1, Fanca, Hmgb3, Igsf3, Mt2, Nop10, Hmgb1, Ahcy, E2f3, Pola2, Srsf5, Hist1h2ao, Hsp90aa1, Gart, Tubb5, Slc39a10, Mycn, Nars, Cdt1, S100a14, Ccnb1, Ncaph, Hmgn2, Tnni1, Fus, Hist1h4n, Ran, Eif2s2, Rpl24, Snora52, Itpa, Mlf1ip, Nhp2, Kcnh1, Hist1h4b, Rpl38, Set, Anp32e, Rps12, Gnl2, Krt16, Snrnp70, Suv39h1, Rps12, Uqcr11, Ptma, E2f8, Hnrnpa1, Tpx2, Hist1h2br, Erdr1, Cdh3, Pbk, Ccnb1, Cdc25a, Hnrnpf, Snora3, Taf1d, Nup133, Scarna17, Rps27a, Knstrn, Tacc3, Snrpg, Kpnb1, Slc7a1, Asf1b, Hist1h2ap, Cks2, Taf1d, Lama5, C1qbp, NA, Ndc1, Hist1h2bm, Pold2, Rps29, Wdr43, Rps29, Mapk6, Ass1, Gm3579, Impdh2, Porcn, Rpl15, Sfn, Rn4.5s, Rps29, Ube2i, Foxm1, Sfn, NA, Plk1, Pla2g4e, Exo1, Pfas, Dgat1, Acta2, Ssrp1, Hif1a, Hspe1, Cep55, Nudc, Cirh1a, Rps27a, Defb3, Hmgb2, Ifrd2, Chaf1a, Kif18b, Snrpd3, Hnrnpa3, Krt6a, Cks2, Hist1h2bb, Glt1d1
**NON-REDUNDANT (Down in Vav2^{Onc})**
4833423E24Rik, Sort1, Spns2, Zdhhc9, Chp2, Nfatc2, F830045P16Rik, Ide, Mfge8, Arhgef5, Lpcat2, Slc6a20a, Blnk, Slc40a1, Il34, Mcc, Zbtb20, Fgf22, Cyp2b10, Efhd1, Tmprss13, Ccr9, Cldn1, Csprs, Wdr45, Gabarapl2, N28178, Carns1, Rnf144b, Ndrg2, Otub2, Cpeb4, D7Ertd443e, Ace2, Gdpd1, Ppp1r1a, Peg3, Abhd14b, Ear1, Usp54, Islr, Alb, Lims2, Slc19a2, March3, Il6ra, Igfbp5, Rab3d, Plcxd1, Tuft1, Ocln, Clic3, Fhdc1, Il1f9, Tmem65, Slc48a1, Tmem54, Bsdc1, Slc5a1, Slc5a8, Nipal1, Wdr65, Map3k9, Gm14137, Tcp11l2, Pnpla1, Dsg1a, 2610206C17Rik, Pak3, Mapk13, Slc4a4, Znf41-ps, Tap1, Tmem184a, Decr2, Gm7609, Adamts12, Srgap2, Myoc, Angptl2, Gm16515, Ripk2, Mup11, Gabarapl1, Map3k8, Chp1, Spsb3, Slc25a42, Blmh, Tmem50b, Mmd, Smim14, Btbd3, Ptk6, Il22ra1, Wfdc5, Ahr, Crct1, Cyb5r4, St3gal5, Appl2, Fam57a, Adhfe1, Ceacam19, Fabp3, Smtnl2, Rnf39, Dgat2, Tmem254a, Ank, AA987161, Gbp8, Sbsn, Klf3, Elovl1, Adipor2, Klk5, Fabp3, Laptm4b, Pla2g2f, Cped1, Tec, Fgf18, Tmem140, Smpdl3a, Cldn23, Hspa12a, Slc35f5, Cmah, Frmd4b, Mup2, Cfd, Csprs, Ssfa2, Gstm2, Cd3g, Mreg, H2-K1, Rhbg, Krt80, Tmem43, Adamts2, Dmkn, Defb6, Ech1, Npr2, Lifr, Stk10, Gstk1, Scnn1a, Dapk1, Lmbrd1, Itih5, Nrep, Mfsd6, Slc37a1, Pcsk6, 2310002J15Rik, Dusp22, Slc22a23, Col14a1, Arpc5, Vsig10l, Hr, Traf6, Fzd4, Krtap17-1, Prdm1, 2010109I03Rik, Gm94, Eps8l2, Mup1, Ggh, Tead1, Slc6a14, Dpep1, Acap2, Kif21a, Ubl7, Ly6g6c, Fchsd2, Mt4, Gbp4, Wfdc3, Csnk2a2, Ccdc80, Rnase6, Smpd1, Man1a, Chl1, Gas7, Serpina3c, Mup2, Aqp7, Zfp772, Dsc1, Cpt1a, Lama2, Enpep, Gm13177, Cd34, Ankrd44, Tprgl, Epcam, Tmem177, Pamr1, Mustn1, Sema4a, Ccl19, NA, Akr1c18, Hmcn1, Slc39a2, Ube4b, Cyb5r4, Add3, Lce3c, Asprv1, Rasgrp3, Pycard, Mapre2, Nlrc5, Gpx3, Slc7a10, Egln3, Slc26a7, 8430408G22Rik, Slc41a3, Dnajb9, Hmcn1, Ear1, Psen1, Zfp52, Gm2a, Gramd3, Gjb3, Mup2, Asah1, Dock11, Neu2, Gpsm1, Prss27, Lmbr1l, Serinc3, Igfbp6, Ang, Hs3st6, Gstm4, Desi1, Hpse, Gstm5, Prkar2b, Cdsn, Serpina3h, Pglyrp4, Smap2, Cpa3, Pgr, Csrp3, Nrn1, Igsf10, Lmcd1, Slc46a1, Abcd2, Ear10, Lpl, 4930429B21Rik, Ghr, Ttpal, Krt84, Ppap2b, Kctd5, Cd82, Hspb1, Fam89a, Dnase1l3, Adamtsl1, sept-08, Cnfn, Nipal4, Palld, Plb1, March8, March2, Tlr11, Ltc4s, Itm2b, Plb1, Ctsa, Nt5c2, Sh3bgrl2, Cln8, Tmem40, Daglb, Chic2, Dusp14, Ndfip1, Ubl3, Klhl13, Htra3, Arsb, Ube2r2, Epn3, Mamdc2, Sh3kbp1, Rassf4, Agpat9, Sts, Hebp2, Cst3, Plin1, Bank1, Rnf208, Tpra1, Tmem50a, Btc, Spink5, Rnf223, Igsf10, Gsn, Kdelr3, Fcna, Timp2, Klf4, H2-T9, Lce1e, Agtr1a, Lynx1, Fn1, 2310022B05Rik, Rps2, Tspan15, Olfr115, Cited4, Paqr8, Arsb, Ccdc120, Guk1, Tgm5, Hspb1, Ttc9, Gltp, Grhl3, Cpq, Sphk1, Lypd5, Gm13139, Micu1, Hmcn1, Ppif, Rab25, Ugcg, Kcnk6, Scamp1, NA, Prcp, Nlrc5, Npas2, Slc45a4, Lipe, Pttg1ip, Cidec, Gstt1, Nlrc5, Gna15, Adipoq, Csprs, Mgl2, Nlrc5, Gng12, Socs2, Fxyd6, Npl, Lgalsl, Trex2, Sepp1, Pcolce, Fuca1, Cygb, Gas6, Cd7, Krtap3-2, Bnip3, Hmcn1, Lyz1, Slc43a2, Htra1, Necap2, Pim1, Timd4, Pld3, Lsp1, Ctsk, Scamp2, Sparcl1, Anpep, Cd1d1, Thbs2, Man1c1, Gm9992, Sh3bp5, Man2b2, Gsta2, Steap3, Kctd11, Tapbp, Tmem141, Arrdc4, Mall, Retn, Mfsd5, Cd209g, Ldb2, Smoc1, Aph1b, Klb, Wfdc18, Mcu, Mapk3, Hexb, Ifi30, Dguok, Ctsh, Irak3, Pcdh1, Rgs18, Sncg, Arntl, Hmcn1, Cd209b, Lum, Dlc1, Gm13306, Hdac9, Pltp, Prkx, Ppp1r27, Slamf9, Nrtn, Tgfbr2, Cbr2, Naaa, Cldn4, Cysltr1, Trf, Cd209f, Mup2, Ngfr, Ebf1, Vgll3, Cercam, Dhrs9, Rgs7, Ubtd1, Tgm1, Fbn1, Kctd12b, Tek, Ephx2, Fcrls, Rgs10, Serpinf1, Nov, Mmp2, Ctso, Scg3, Igsf10, H2-T10, Dpt, Sh3bgrl, Chst15, Plekha2, Gpr133, Rnf11, Pmp22, Klk7, Mal2, Cd248, Kctd12,

Tmeff2, Rab11fip1, Scn7a, Klk8, Nlrc5, Gypc, Mrgprb1, Man2b1, Sprr1a, Greb1, Plk3, Dok2, Cd209c, Scara3, Ctgf, Itpkb, Camk2n1, Gm11564, H2-M3, Mmp11, Col15a1, Atp6v0d1, Fcgrt, Prkcb, Arrb1, Glrx, Il1a, Hmcn1, Pnp2, H2-Q4, Irgm2, Prex2, Tm6sf1, Mnf1, Pla2g2d, Ifi203, Chac1, Sgce, Gng4, Psmb9, Ctsl, Prss23, Abcg1, Tcn2, Irak2, Aoah, Tbxas1, Dcn, Car6, Iigp1b, Csf1r, Ccl19, Ly6d, Serping1, Ndn, Plod2, Wisp2, C3, Plac9a, Fabp4, Reln, Cd209a, Cmklr1, Hsd17b11, Calcrl, Nid1, Laptm5, Cmtm3, Cybb, Dkk2, Mxra8, Loxl1, Serpina3f, Colec12, Sprr4, Tgm2, Pi16, Fstl1, Cd163, Gbp6, Degs2, Abtb2, Dpysl3, Lox, Gm4951, Loxl2, Krtap5-1, Slc5a3, Spon1, Stard8, Tgtp1, Slc43a3, Ecm1, Itga4, 9930111J21Rik2, Gbp7, Pxdn, Sp100, Irgm1, Blvrb, Igfbp3, Defb1, Mcpt4, Ccl19, Gbp9, Mxd1, Cdh11, C1s, Klk10, C1qtnf1, Vsig4, Tgtp1, Igf1, Plb1, Il1r2, Qpct, Cfhr2, Gm5431, Lpgat1, Psmb10, Ddx60, Gm10639, Cd37, Rhoj, Krtap5-4, AI607873, AW551984, Ntrk2, Gfpt2, Gatm, Cfh, Lyz2, Rac2, Ly6g6e, Alox5, C1qa, Svep1, Rassf2, F13a1, Fam26e, Dock2, Dab2, Ndrg1, Cd48, Pgm1, Plac9a, Ptplad2, Gbgt1, Lgals3bp, Tlr7, Xist, Krtap1-3, Mrc1, Pld4, C1qc, Tshr, Sfrp2, Anxa3, Gbp2, Krtap4-13, Ms4a8a, Efemp1, C1rb, Gvin1, Emr1, Ggt5, Gpc3, Emp3, Ube2l6, Cyp1b1, Folr2, Slc16a12, Unc93b1, C1qb, Siglec1, Tpsb2, Pla1a, Lbp, Sdc3, Mest, Ednrb, Nrp1, Sfrp4, Mfap5, Osr1, Fcgr2b, Cd68, Selplg, C1ra, Krtap9-3, Iigp1, Cfp, Trim30a, Gabra3, Ly86, Lyve1, Krtap4-7, Fcer1g, Cd209e, Ccl21a, Gabrp, Oasl2, Ifi205, Mmrn1, Pf4, Fcgr3, AW112010, Ccl8, Krtap4-2, Sod3, Gsta1, P2ry13, Pinlyp, Krtap2-4, Krtap9-1, Chrdl1, Cd209d, Ifit3, Gm12250, Clec10a, Lcp1, Snord118, Nnmt, Ccl11, Krtap4-13, Gm11563, Krtap4-6, Cd38, Krtap2-4, Cp, Alox5ap, Angptl4, Wfdc17, Lep, Plac8, Ccl6, Krtap4-1, Krtap1-5, Gm11567, Ccl9, Gm10228, Mup7, Krtap21-1, Krtap4-16, Ifitm6, Krtap6-2, Krtap6-5, Krtap6-1, Cxcl13, Prr9, Krtap7-1

SUPPLEMENTARY TABLE 3. List of genes belonging to the Vav2^{Onc}-Sig.

GENE SYMBOL	PATTERN IN Vav2^{Onc}	PATTERN IN *Vav2*$^{-/-}$;*Vav3*$^{-/-}$ + TPA	PATTERN IN cSCC	PATTERN IN ORAL SCC
AREG	up	down	up	up
AURKA	up	down	up	up
AURKB	up	down	up	up
BCAT1	up	down	up	up
BIRC5	up	down	up	up
BNC1	up	down	up	up
CCL4	up	down	up	up
CCT5	up	down	up	up
CDC20	up	down	up	up
CDCA5	up	down	up	up
CDCA8	up	down	up	up
CDK1	up	down	up	up
CENPN	up	down	up	up
CKS1B	up	down	up	up
CKS2	up	down	up	up
CXCL2	up	down	up	up
E2F7	up	down	up	up
ENAH	up	down	up	up
FEN1	up	down	up	up
FOSL1	up	down	up	up
FSCN1	up	down	up	up
HAS3	up	down	up	up
HMGB3	up	down	up	up
IFITM1	up	down	up	up
IL1B	up	down	up	up
IL24	up	down	up	up
INHBA	up	down	up	up
ITGB1	up	down	up	up
KPNA2	up	down	up	up
LAMC2	up	down	up	up
LIPG	up	down	up	up
LRP8	up	down	up	up
MCM10	up	down	up	up
MMP13	up	down	up	up
MMP9	up	down	up	up
MTHFD2	up	down	up	up
MYC	up	down	up	up
NDC80	up	down	up	up
NEK2	up	down	up	up
NOLC1	up	down	up	up
NRG1	up	down	up	up
NRP2	up	down	up	up
ODC1	up	down	up	up
PDPN	up	down	up	up
PI15	up	down	up	up
PLAUR	up	down	up	up
PLEK	up	down	up	up
PTGS2	up	down	up	up
RAD51AP1	up	down	up	up
RANBP1	up	down	up	up

GENE SYMBOL	PATTERN IN Vav2^{Onc}	PATTERN IN *Vav2*$^{-/-}$;*Vav3*$^{-/-}$ + TPA	PATTERN IN cSCC	PATTERN IN ORAL SCC
RRM2	up	down	up	up
SERPINE1	up	down	up	up
SH2D5	up	down	up	up
SLC20A1	up	down	up	up
SLC2A3	up	down	up	up
SLC7A11	up	down	up	up
SLC7A5	up	down	up	up
SOCS3	up	down	up	up
SPP1	up	down	up	up
SRM	up	down	up	up
STOM	up	down	up	up
TK1	up	down	up	up
TNC	up	down	up	up
TREM1	up	down	up	up
TRIP13	up	down	up	up
UBE2S	up	down	up	up
ABCA5	down	up	down	down
ABCA6	down	up	down	down
ABCA9	down	up	down	down
ACPP	down	up	down	down
ADSSL1	down	up	down	down
AFF3	down	up	down	down
ALDH1A1	down	up	down	down
ANKRD35	down	up	down	down
ANXA9	down	up	down	down
AR	down	up	down	down
ARHGAP20	down	up	down	down
ATP12A	down	up	down	down
ATP1A2	down	up	down	down
ATP1B1	down	up	down	down
BBOX1	down	up	down	down
CAMK1D	down	up	down	down
CARD14	down	up	down	down
CDO1	down	up	down	down
CDON	down	up	down	down
CGN	down	up	down	down
CILP	down	up	down	down
COBL	down	up	down	down
CUL3	down	up	down	down
DLG2	down	up	down	down
EXPH5	down	up	down	down
FAM189A2	down	up	down	down
FAM3B	down	up	down	down
FMO2	down	up	down	down
FXYD1	down	up	down	down
FYCO1	down	up	down	down
GAN	down	up	down	down
GRAMD1C	down	up	down	down
HMGCS2	down	up	down	down
HSPB6	down	up	down	down
IL18	down	up	down	down
IL20RA	down	up	down	down

GENE SYMBOL	PATTERN IN Vav2^{Onc}	PATTERN IN *Vav2*$^{-/-}$;*Vav3*$^{-/-}$ + TPA	PATTERN IN cSCC	PATTERN IN ORAL SCC
INMT	down	up	down	down
ITM2A	down	up	down	down
KRT15	down	up	down	down
LDB3	down	up	down	down
LNX1	down	up	down	down
LRRN4CL	down	up	down	down
MAOB	down	up	down	down
MBOAT2	down	up	down	down
MFAP4	down	up	down	down
MYOZ1	down	up	down	down
NEBL	down	up	down	down
OGN	down	up	down	down
PCOLCE2	down	up	down	down
PKIA	down	up	down	down
PKIB	down	up	down	down
POF1B	down	up	down	down
PYGM	down	up	down	down
RECK	down	up	down	down
RORA	down	up	down	down
SCARA5	down	up	down	down
SCEL	down	up	down	down
SH3RF2	down	up	down	down
SLC16A14	down	up	down	down
SLC24A3	down	up	down	down
SLC2A12	down	up	down	down
SLC6A4	down	up	down	down
SLURP1	down	up	down	down
SOX6	down	up	down	down
SSBP2	down	up	down	down
TCEA3	down	up	down	down
TJP3	down	up	down	down
TPPP	down	up	down	down
TPPP3	down	up	down	down
TRIM7	down	up	down	down

SUPPLEMENTARY TABLE 4. List of genes belonging to the minimal Vav2^{Onc} diagnostic gene signature

GENE SYMBOL	PATTERN IN Vav2^{Onc}	PATTERN IN *Vav2*$^{-/-}$;*Vav3*$^{-/-}$ + TPA	PATTERN IN cSCC	PATTERN IN ORAL SCC
AREG	up	down	up	up
AURKA	up	down	up	up
BCAT1	up	down	up	up
BIRC5	up	down	up	up
CCL4	up	down	up	up
CDCA5	up	down	up	up
CDCA8	up	down	up	up
CKS1B	up	down	up	up
CXCL2	up	down	up	up
FSCN1	up	down	up	up
HAS3	up	down	up	up
IL24	up	down	up	up
LAMC2	up	down	up	up
MMP9	up	down	up	up
MYC	up	down	up	up
NEK2	up	down	up	up
NOLC1	up	down	up	up
ODC1	up	down	up	up
SERPINE1	up	down	up	up
SLC20A1	up	down	up	up
SLC2A3	up	down	up	up
SLC7A11	up	down	up	up
SLC7A5	up	down	up	up
SOCS3	up	down	up	up
SRM	up	down	up	up
ABCA9	down	up	down	down
ACPP	down	up	down	down
ANXA9	down	up	down	down
ARHGAP20	down	up	down	down
ATP1A2	down	up	down	down
CAMK1D	down	up	down	down
CILP	down	up	down	down
EXPH5	down	up	down	down
FYCO1	down	up	down	down
HSPB6	down	up	down	down
PKIB	down	up	down	down
PYGM	down	up	down	down
SCEL	down	up	down	down
SLC6A4	down	up	down	down
SSBP2	down	up	down	down
TCEA3	down	up	down	down

SUPPLEMENTARY TABLE 5. List of patient samples used in this study

SAMPLE ID	SAMPLE TYPE	SOURCE
B87	Healthy	University Central Hospital of Asturias
B100	Healthy	University Central Hospital of Asturias
B103	Healthy	University Central Hospital of Asturias
B107	Healthy	University Central Hospital of Asturias
B116	Healthy	University Central Hospital of Asturias
B135	Healthy	University Central Hospital of Asturias
B140	Healthy	University Central Hospital of Asturias
B141	Healthy	University Central Hospital of Asturias
B143	Healthy	University Central Hospital of Asturias
B147	Healthy	University Central Hospital of Asturias
B158	Healthy	University Central Hospital of Asturias
B161	Healthy	University Central Hospital of Asturias
B169	Healthy	University Central Hospital of Asturias
B172	Healthy	University Central Hospital of Asturias
B174	Healthy	University Central Hospital of Asturias
B175	Healthy	University Central Hospital of Asturias
B183	Healthy	University Central Hospital of Asturias
T87	hnSCC	University Central Hospital of Asturias
T91	hnSCC	University Central Hospital of Asturias
T92	hnSCC	University Central Hospital of Asturias
T97	hnSCC	University Central Hospital of Asturias
T98	hnSCC	University Central Hospital of Asturias
TL100	hnSCC	University Central Hospital of Asturias
TP100	hnSCC	University Central Hospital of Asturias
T103	hnSCC	University Central Hospital of Asturias
T104	hnSCC	University Central Hospital of Asturias
T106	hnSCC	University Central Hospital of Asturias
T107	hnSCC	University Central Hospital of Asturias
T108	hnSCC	University Central Hospital of Asturias
T109	hnSCC	University Central Hospital of Asturias
T112	hnSCC	University Central Hospital of Asturias
T116	hnSCC	University Central Hospital of Asturias
T119	hnSCC	University Central Hospital of Asturias
T129	hnSCC	University Central Hospital of Asturias
T133	hnSCC	University Central Hospital of Asturias
T135	hnSCC	University Central Hospital of Asturias
T140	hnSCC	University Central Hospital of Asturias
T141	hnSCC	University Central Hospital of Asturias
T143	hnSCC	University Central Hospital of Asturias
T147	hnSCC	University Central Hospital of Asturias
T151	hnSCC	University Central Hospital of Asturias
T155	hnSCC	University Central Hospital of Asturias
T158	hnSCC	University Central Hospital of Asturias
T161	hnSCC	University Central Hospital of Asturias
T168	hnSCC	University Central Hospital of Asturias

SAMPLE ID	SAMPLE TYPE	SOURCE
T169	hnSCC	University Central Hospital of Asturias
T172	hnSCC	University Central Hospital of Asturias
T174	hnSCC	University Central Hospital of Asturias
T175	hnSCC	University Central Hospital of Asturias
T183	hnSCC	University Central Hospital of Asturias
S12	Healthy	Sant Pau i Santa Creu Hospital
S139	Healthy	Sant Pau i Santa Creu Hospital
S463	Healthy	Sant Pau i Santa Creu Hospital
S524	Healthy	Sant Pau i Santa Creu Hospital
S542	Healthy	Sant Pau i Santa Creu Hospital
S548	Healthy	Sant Pau i Santa Creu Hospital
S564	Healthy	Sant Pau i Santa Creu Hospital
S568	Healthy	Sant Pau i Santa Creu Hospital
S743	Healthy	Sant Pau i Santa Creu Hospital
S1039	Healthy	Sant Pau i Santa Creu Hospital
T11	hnSCC	Sant Pau i Santa Creu Hospital
T138	hnSCC	Sant Pau i Santa Creu Hospital
T289	hnSCC	Sant Pau i Santa Creu Hospital
T306	hnSCC	Sant Pau i Santa Creu Hospital
T334	hnSCC	Sant Pau i Santa Creu Hospital
T462	hnSCC	Sant Pau i Santa Creu Hospital
T523	hnSCC	Sant Pau i Santa Creu Hospital
T541	hnSCC	Sant Pau i Santa Creu Hospital
T547	hnSCC	Sant Pau i Santa Creu Hospital
T563	hnSCC	Sant Pau i Santa Creu Hospital
T567	hnSCC	Sant Pau i Santa Creu Hospital
T742	hnSCC	Sant Pau i Santa Creu Hospital
T1038	hnSCC	Sant Pau i Santa Creu Hospital
T27598	hnSCC	University Hospital of Salamanca
T27629	hnSCC	University Hospital of Salamanca
T27767	hnSCC	University Hospital of Salamanca
T27824	hnSCC	University Hospital of Salamanca
T28160	hnSCC	University Hospital of Salamanca
T28252	hnSCC	University Hospital of Salamanca
T28288	hnSCC	University Hospital of Salamanca
T29277	hnSCC	University Hospital of Salamanca
T29331	hnSCC	University Hospital of Salamanca
T29894	hnSCC	University Hospital of Salamanca

SUPPLEMENTARY NOTE 1. Genomic characterization of the hnSCC patient-derived cells used in this study

Using next generation sequencing (see Methods), we found that the VdH01 and VdH015 patient-derived cells are clearly different according to the catalogue of both deleterious single nucleotide (**Supplementary Fig. 6a**) and copy number (**Supplementary Fig. 6b,c**) variations present in them. These disparities are expected, given that each of those cells has been obtained from an independent patient. The two patient-derived cells share, however, alterations that target common loci (*APOBEC3B*, *AXIN1*, *BCOR*, *CASP8*, *CREBBP*, *DDX3X*, *EP300*, *FAT1*, *FHIT*, *GRIN2A*, *MRTFA*, *MYH9*, *NTHL1*, *ROBO2*, *TP53*, *TRAF7*, *TSC2*, *ZRSR29*) (**Supplementary Fig. 6a-c**). Mutations in *FAT1*, *TP53*, and *ROBO2* are also present in the SCC-25 cancer cell line (**Supplementary Fig. 6a-c**). The two patient-derived cells are also HPV^{-} (**Fig. 6a**) and show a gain in the long arm of chromosome 9, a frequent event in hnSCC from oral regions[1, 2]. Many of the genetic alterations found in each of those patient-derived cells have been previously detected at high frequency rates in hnSCC cases (*CASP8*, *CDKN2A*, *EP300*, *FAT1*, *NOTCH1*, *MYC*, *PABPC1*, *RAD21*, *RECQL4*, *TERT*, *SPEN*, *TP53*) (**Supplementary Fig. 6a-d**). In the case of *VAV2*, we did not find any activating single point mutation in any of the two patient-derived cells (**Supplementary Fig. 6a**). The VdH15 cells do bear a small deletion of 1,068 bp in a region downstream of the *VAV2* locus that harbors a putative binding site for the transcriptional factor ZNF263 (**Supplementary Fig. 6e**; coordinates chr9:136625194-136626262), although the functional relevance of this alteration for either *VAV2* expression or hnSCC etiology is unknown. Interestingly, we found a point mutation in *VAV1* (Q122R) in VdH01 patient-derived cells. An analogous mutation (Q122H) has been found before in a cervical SCC case in TCGA studies. These mutations, however, are unlikely to create a catalytic gain-of-function event on the protein according to previous functional and structural studies[3, 4]. The VdH01 cells also show amplification events affecting *PREX2* and *ARHGAP5*, two loci encoding a RAC1 GEF and RHOA GTPase activating protein, respectively. Although paradoxical from a functional point of view, the amplification of the latter gene has been observed before in a number of tumors[5].

SUPPLEMENTARY REFERENCES

1. Martin, C.L. *et al.* Chromosomal imbalances in oral squamous cell carcinoma: examination of 31 cell lines and review of the literature. *Oral Oncol* **44**, 369-382 (2008).

2. Pickering, C.R. *et al.* Integrative genomic characterization of oral squamous cell carcinoma identifies frequent somatic drivers. *Cancer Discov* **3**, 770-781 (2013).

3. Llorca, O., Arias-Palomo, E., Zugaza, J.L. & Bustelo, X.R. Global conformational rearrangements during the activation of the GDP/GTP exchange factor Vav3. *EMBO J* **24**, 1330-1340 (2005).

4. Yu, B. *et al.* Structural and energetic mechanisms of cooperative autoinhibition and activation of Vav1. *Cell* **140**, 246-256 (2010).

5. Héraud, C., Pinault, M., Lagrée, V. & Moreau, V. The p190RhoGAPs. *Cells* **8** (2019).
