# Supplementary material for: VAV2 signaling promotes regenerative proliferation in both cutaneous and head and neck squamous cell carcinoma
Source: Nat Commun. 2020 Sep 22;11:4788. doi: 10.1038/s41467-020-18524-3 (PMC7508832; doi:10.1038/s41467-020-18524-3)
Supplement: Supplementary file 3 — Description of Additional Supplementary Files [file 41467_2020_18524_MOESM3_ESM.pdf]

Description of Additional Supplementary Files

File Name: Supplementary Data 1
Description: List of genes belonging to the Vav2Onc-regulated transcriptome.
