# Supplementary material for: VAV2 signaling promotes regenerative proliferation in both cutaneous and head and neck squamous cell carcinoma
Source: Nat Commun. 2020 Sep 22;11:4788. doi: 10.1038/s41467-020-18524-3 (PMC7508832; doi:10.1038/s41467-020-18524-3)
Supplement: Supplementary file 5 — Reporting Summary [file 41467_2020_18524_MOESM5_ESM.pdf]

nature research

Corresponding author(s): Xosé R. Bustelo

Last updated by author(s): Aug 17, 2020

Reporting Summary

Nature Research wishes to improve the reproducibility of the work that we publish. This form provides structure for consistency and transparency in reporting. For further information on Nature Research policies, see Authors & Referees and the Editorial Policy Checklist .

Statistics

For all statistical analyses, confirm that the following items are present in the figure legend, table legend, main text, or Methods section.

n/a	Confirmed	
☐	☒	The exact sample size (*n*) for each experimental group/condition, given as a discrete number and unit of measurement
☐	☒	A statement on whether measurements were taken from distinct samples or whether the same sample was measured repeatedly
☐	☒	The statistical test(s) used AND whether they are one- or two-sided<br>*Only common tests should be described solely by name; describe more complex techniques in the Methods section.*
☐	☒	A description of all covariates tested
☐	☒	A description of any assumptions or corrections, such as tests of normality and adjustment for multiple comparisons
☐	☒	A full description of the statistical parameters including central tendency (e.g. means) or other basic estimates (e.g. regression coefficient) AND variation (e.g. standard deviation) or associated estimates of uncertainty (e.g. confidence intervals)
☐	☒	For null hypothesis testing, the test statistic (e.g. *F*, *t*, *r*) with confidence intervals, effect sizes, degrees of freedom and *P* value noted<br>*Give P values as exact values whenever suitable.*
☒	☐	For Bayesian analysis, information on the choice of priors and Markov chain Monte Carlo settings
☒	☐	For hierarchical and complex designs, identification of the appropriate level for tests and full reporting of outcomes
☐	☒	Estimates of effect sizes (e.g. Cohen's *d*, Pearson's *r*), indicating how they were calculated

Our web collection on statistics for biologists contains articles on many of the points above.

Software and code

Policy information about availability of computer code

Data collection	No software was used for data collection.
Data analysis	Open-source and R/Bioconductor software packages: RMA (affy version 1.64.0), fRMA (version 1.40.0), Limma (version 3.42.2), TCGAbiolinks (version 2.17.3), Survival (version 3.2.3), Heatmap3 (version 1.1.7), OmicCircos (version 1.26.0). ImageJ (version 2.0.0-rc-69/1.52p), iRegulon (version 1.3), DAVID (version 6.8), ToppFun (version 1.0), GSEA (version 4.1.0), ssGSEA (version 2.0).

For manuscripts utilizing custom algorithms or software that are central to the research but not yet described in published literature, software must be made available to editors/reviewers. We strongly encourage code deposition in a community repository (e.g. GitHub). See the Nature Research guidelines for submitting code & software for further information.

Data

Policy information about availability of data

All manuscripts must include a data availability statement. This statement should provide the following information, where applicable:

- Accession codes, unique identifiers, or web links for publicly available datasets
- A list of figures that have associated raw data
- A description of any restrictions on data availability

Microarray data that support the findings of this study have been deposited in GEO database with the accession code GSE124019. In addition, GEO datasets GSE45216, GSE13355, GSE30784, GSE21264, GSE52651, GSE26713 and the TCGA HNSC, CESC and LUSC datasets were used to interrogate VAV2 enrichment levels. Survival analyses were performed using the GSE41613, GSE42743, and the TCGA HNSC datasets. GSEA was performed using the datasets GSE52954, GSE29328, GSE10423, GSE5576, GSE72939 and GSE66083.

Field-specific reporting

Please select the one below that is the best fit for your research. If you are not sure, read the appropriate sections before making your selection.

- [x] Life sciences
- [ ] Behavioural & social sciences
- [ ] Ecological, evolutionary & environmental sciences

For a reference copy of the document with all sections, see nature.com/documents/nr-reporting-summary-flat.pdf

Life sciences study design

All studies must disclose on these points even when the disclosure is negative.

Sample size	Minimum sample size was estimated using Lehr's equation. At least three independent replicates were performed in all experiments. For experiments subjected to higher variability, such as animal-based studies, at least six biological replicates were performed. The sample size used for each experiment is indicated at the corresponding figure legend in the manuscript.
Data exclusions	No data were excluded.
Replication	At least three independent replicates were performed in all experiments. For experiments subjected to higher variability, such as animal-based studies, at least six biological replicates were performed. The number of independent replicates for each experiment is indicated at the corresponding figure legend in the manuscript.
Randomization	In all cell and animal studies, groups were allocated randomly.
Blinding	For all animal studies, the investigators were blind to group allocation. Blinding was not applicable to the rest of experiments.

Reporting for specific materials, systems and methods

We require information from authors about some types of materials, experimental systems and methods used in many studies. Here, indicate whether each material, system or method listed is relevant to your study. If you are not sure if a list item applies to your research, read the appropriate section before selecting a response.

Materials & experimental systems

n/a	Involved in the study	
[ ]	[x]	Antibodies
[ ]	[x]	Eukaryotic cell lines
[x]	[ ]	Palaeontology
[ ]	[x]	Animals and other organisms
[ ]	[x]	Human research participants
[x]	[ ]	Clinical data

Methods

n/a	Involved in the study	
[x]	[ ]	ChIP-seq
[x]	[ ]	Flow cytometry
[x]	[ ]	MRI-based neuroimaging

Antibodies

Antibodies used	Vav2 (1:1000 dilution; Homemade; Lab catalog No. 580-2), phospho-tyrosine residues (1:1000 dilution; Santa Cruz Biotechnologies, Catalog No. sc- 7020), GFP (1:5,000 dilution; Covance, Catalog No. MMS-118P), the hemagglutinin (HA) epitope (1:1,000 dilution; Cell Signaling, Catalog No. 3724), Rac1 (1:1,000 dilution; BD Biosciences, Catalog No. 610651), RhoA (1:1,000 dilution; Cell Signaling, Catalog No. 2117), Cdc42 (1:1,000 dilution; Santa Cruz Biotechnology, Catalog No. sc-87), c-Myc (1:1,000 dilution; Merck, Catalog No. 06- 340), and tubulin a (1:2,000 dilution; Calbiochem, Catalog No. CP06), VAV2 (Abcam, Catalog No. ab52640; 1:50 dilution), c-Fos (1:50 dilution, Santa Cruz Biotechnology, Catalog No. sc-166940), c-Myc (1:50 dilution, Abcam, Catalog No. ab32072), YAP (1:200 dilution, Novus Biologicals, Catalog No. NB110-58358), Cyclin D1 (1:200 dilution, Roche, Catalog No. 790-4508), keratin 14 (1:300 dilution, Biolegend, Catalog No. 905301), involucrin (1:100 dilution, Sigma-Aldrich, Catalog No. I9018)
Validation	Commercially-available antibodies (see above) have been validated by the manufacturer for the application (immunoblot or immunohistochemistry) and species (mouse or human) they have been used for in our study. This information is available at each manufacturer's website and can be obtained through the catalog numbers indicated above. The homemade Vav2 antibody has been validated by us in overexpression, knockdown and knockout experiments.

Eukaryotic cell lines

Policy information about cell lines

Cell line source(s)	KerCT and HEK293 (obtained from the ATCC); SCC-25 (ATCC, obtained from Salvador Aznar Benitah), VdH15 and VdH01 (obtained from Salvador Aznar Benitah). iPS cells were generated from mouse primary keratinocytes isolated from the

	animals indicated below.
Authentication	ATCC cell lines are authenticated by the manufacturer. No other authetication was performed.
Mycoplasma contamination	All cell lines tested negative for mycoplasma contamination.
Commonly misidentified lines (See ICLAC register)	No commonly misidentified cell lines were used in this study.

Animals and other organisms

Policy information about studies involving animals; ARRIVE guidelines recommended for reporting animal research

Laboratory animals	Mus musculus, C57BL/6 background, 8-10 week old males. The genotype (Vav2Onc/Onc or WT) and age of the animals used in each experiment is detailed in the Methods section of the manuscript. Animals were kept in ventilated rooms in pathogen–free facilities under controlled temperature (23ºC), humidity (50%), and illumination (12–hour–light/12–hour–dark cycle) conditions.
Wild animals	No wild animals were used in this study.
Field-collected samples	No field-collected samples were used in this study.
Ethics oversight	Bioethics committee of Salamanca University.

Note that full information on the approval of the study protocol must also be provided in the manuscript.

Human research participants

Policy information about studies involving human research participants

Population characteristics	Patients with HPV-negative head and neck squamous cell carcinoma (no age or gender restriction).
Recruitment	Patients with HPV-negative head and neck squamous cell carcinoma (no age or gender restriction). We are not aware of any bias in the recruitment.
Ethics oversight	The use of patient samples was conducted in accordance to the Declaration of Helsinki and approved by the Institutional Ethic Committees of the Institute for Research in Biomedicine, Vall d'Hebron Research Institute, and the Hospital Universitario Central de Asturias.

Note that full information on the approval of the study protocol must also be provided in the manuscript.
