# Supplementary figures and images for: VAV2 signaling promotes regenerative proliferation in both cutaneous and head and neck squamous cell carcinoma
Source: Nat Commun. 2020 Sep 22;11:4788. doi: 10.1038/s41467-020-18524-3 (PMC7508832; doi:10.1038/s41467-020-18524-3)

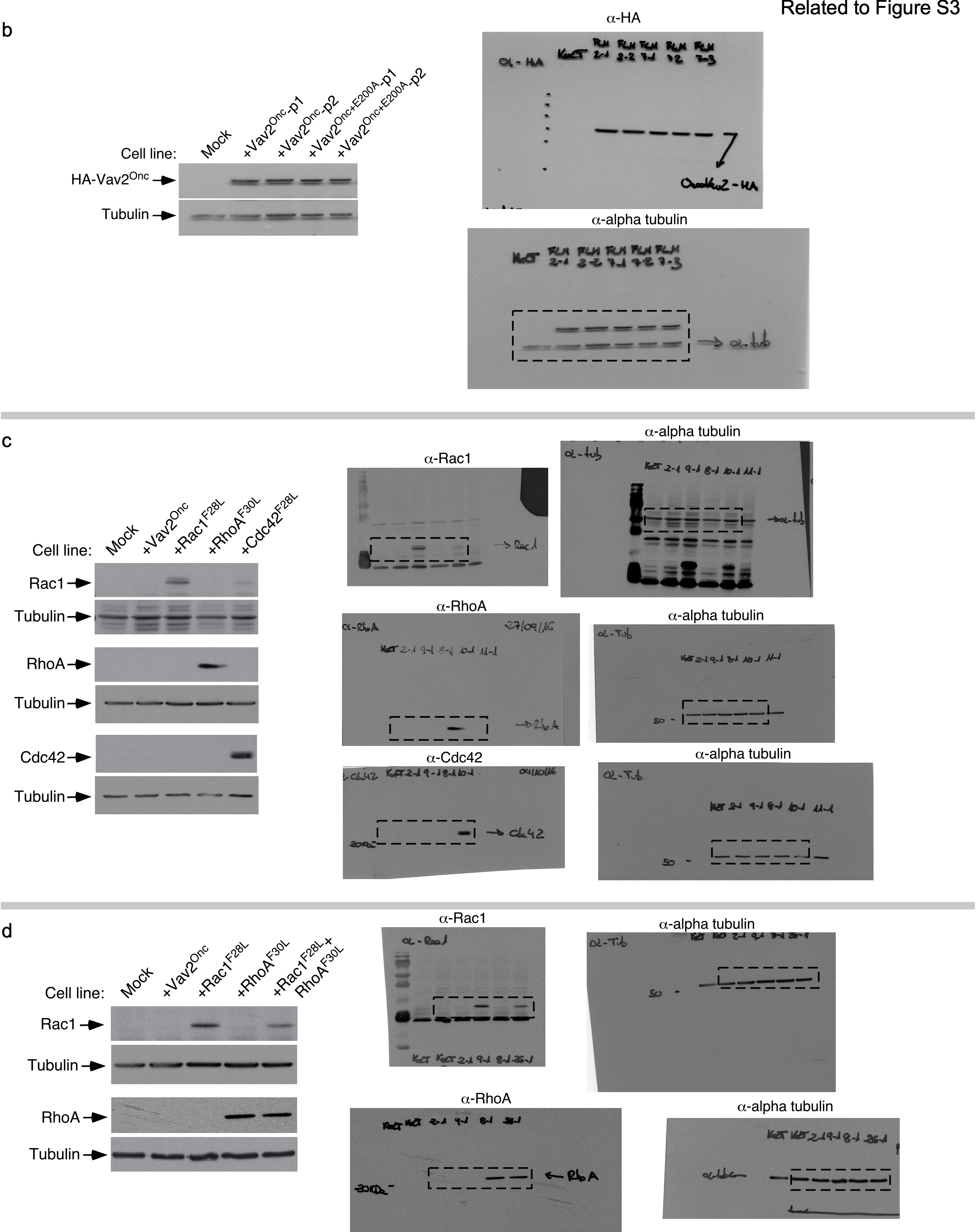

Supplement: Supplementary file 6 — Source Data [file 41467_2020_18524_MOESM6_ESM.zip › 5.4. SOURCE DATA/WB raw data/Figure 3S.Raw.WB.tif]

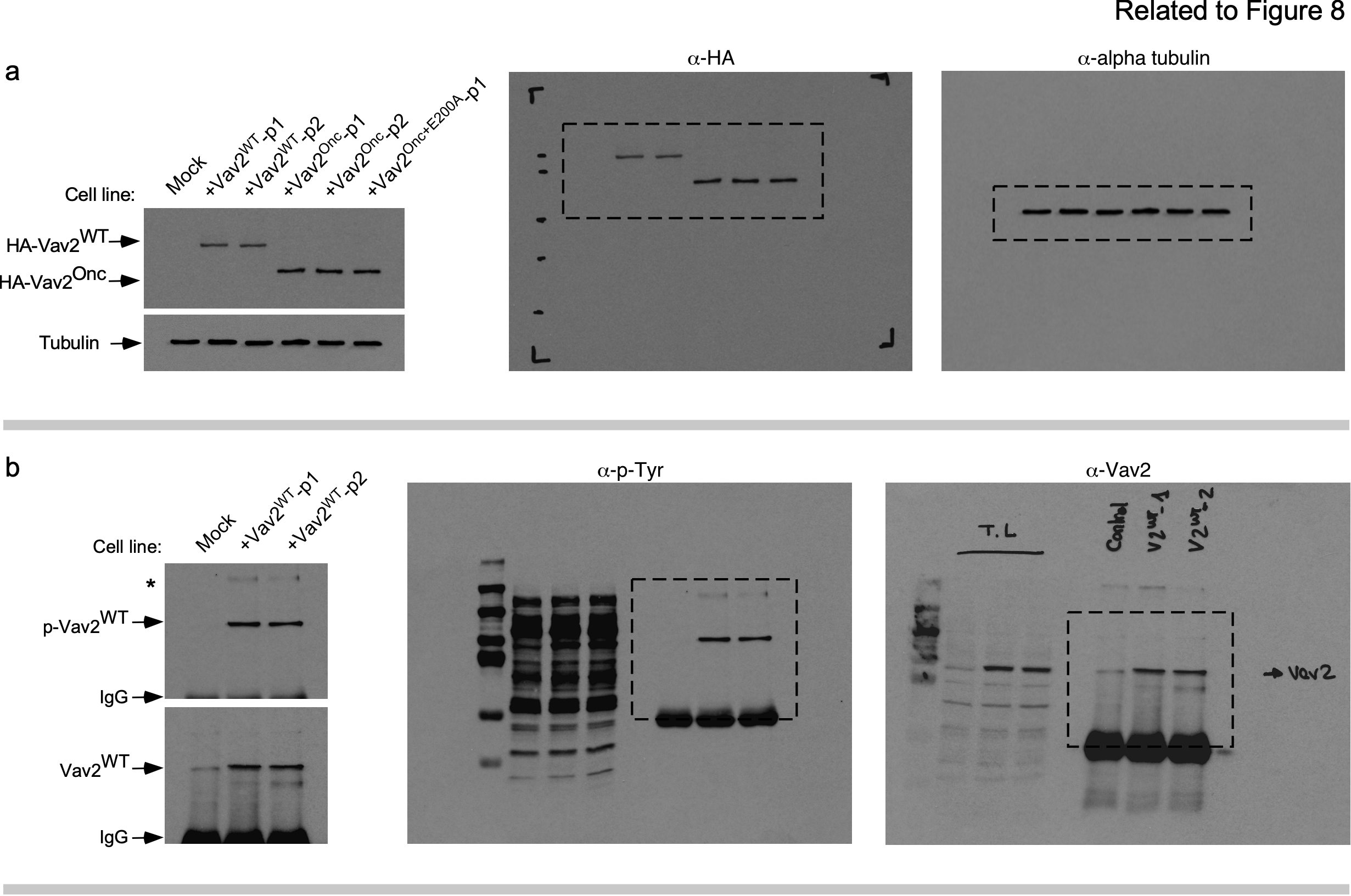

Supplement: Supplementary file 6 — Source Data [file 41467_2020_18524_MOESM6_ESM.zip › 5.4. SOURCE DATA/WB raw data/Figure 8.Raw.WB.tif]

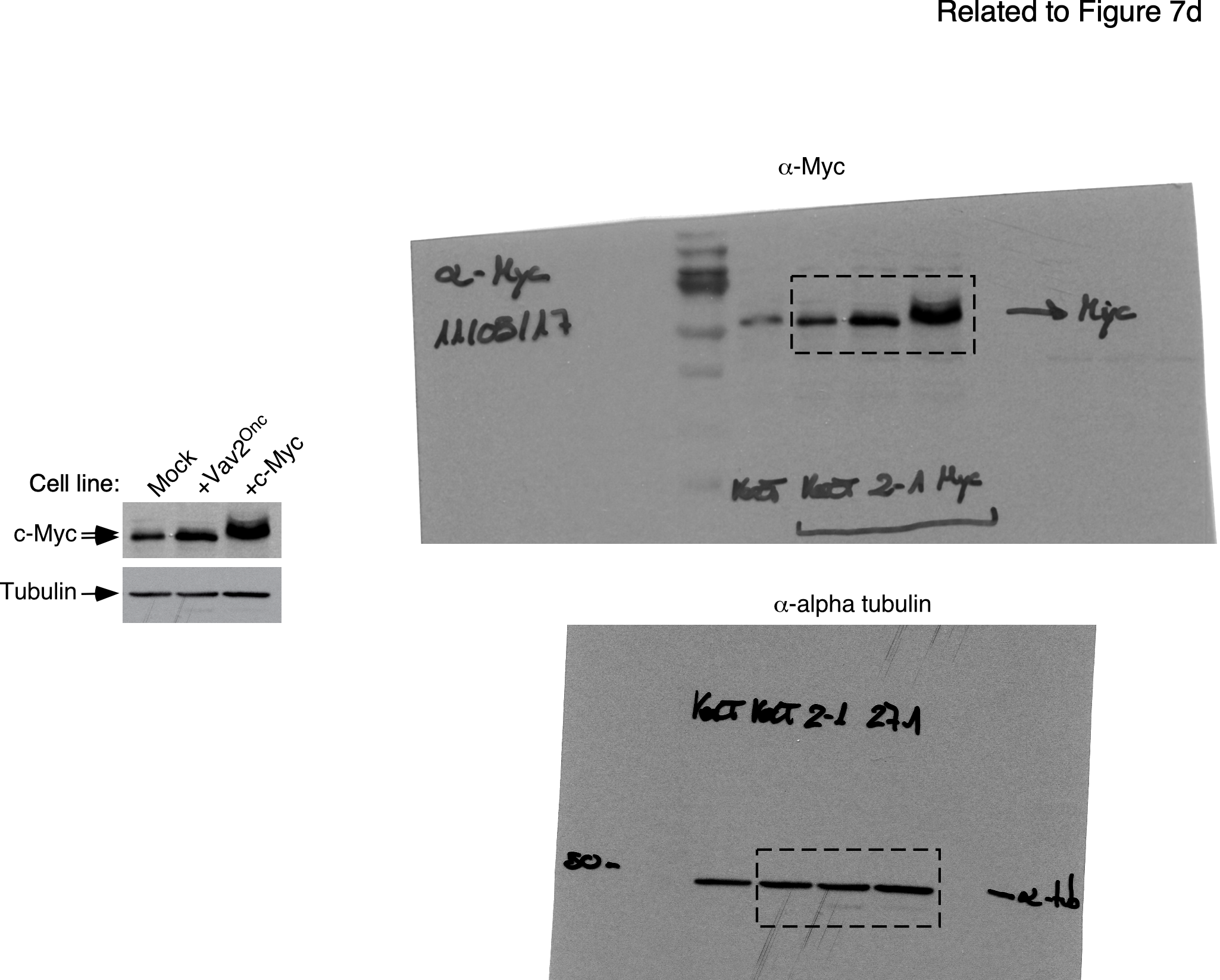

Supplement: Supplementary file 6 — Source Data [file 41467_2020_18524_MOESM6_ESM.zip › 5.4. SOURCE DATA/WB raw data/Figure 7.Raw.WB.tif]

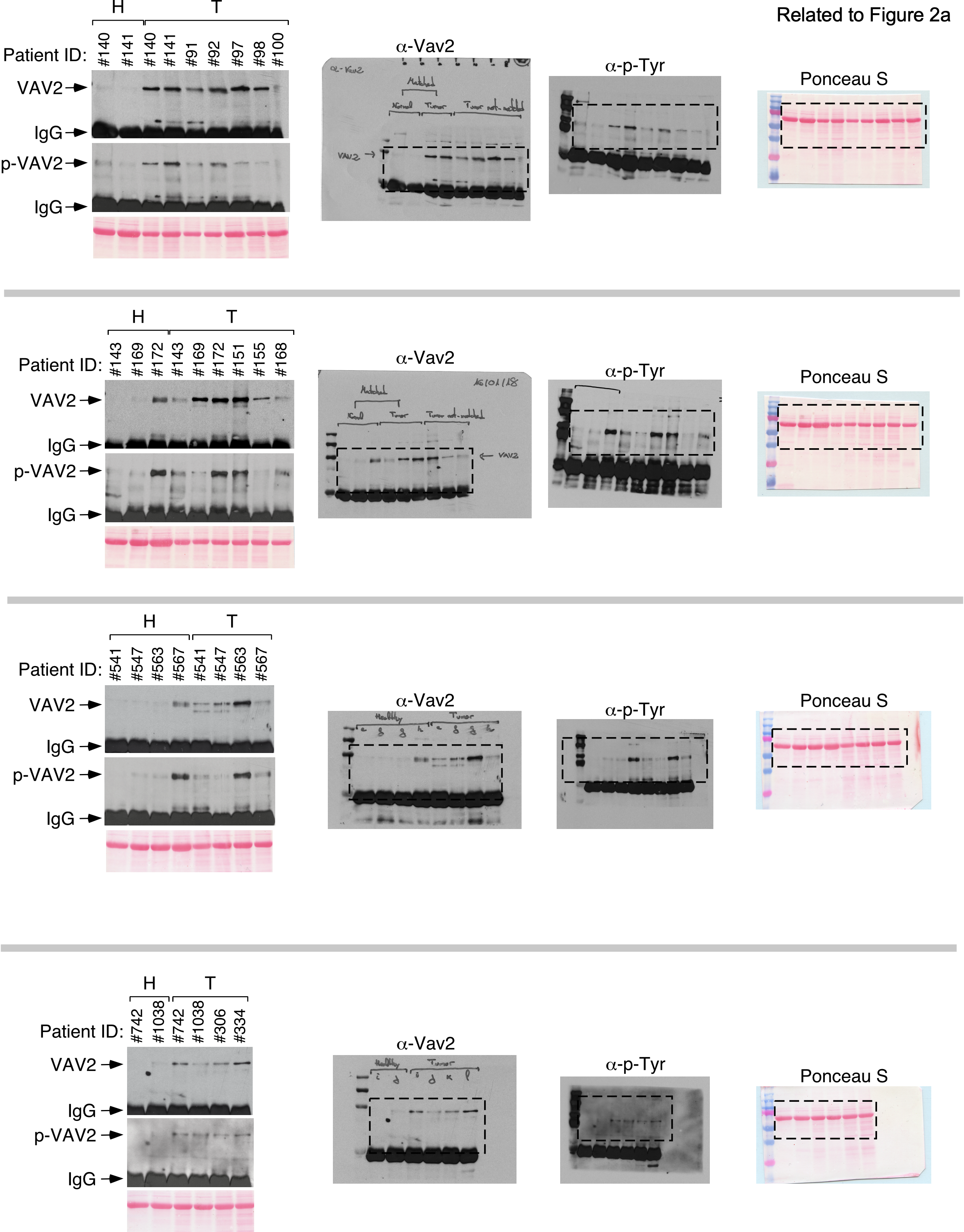

Supplement: Supplementary file 6 — Source Data [file 41467_2020_18524_MOESM6_ESM.zip › 5.4. SOURCE DATA/WB raw data/Figure 2.Raw.WB.tif]

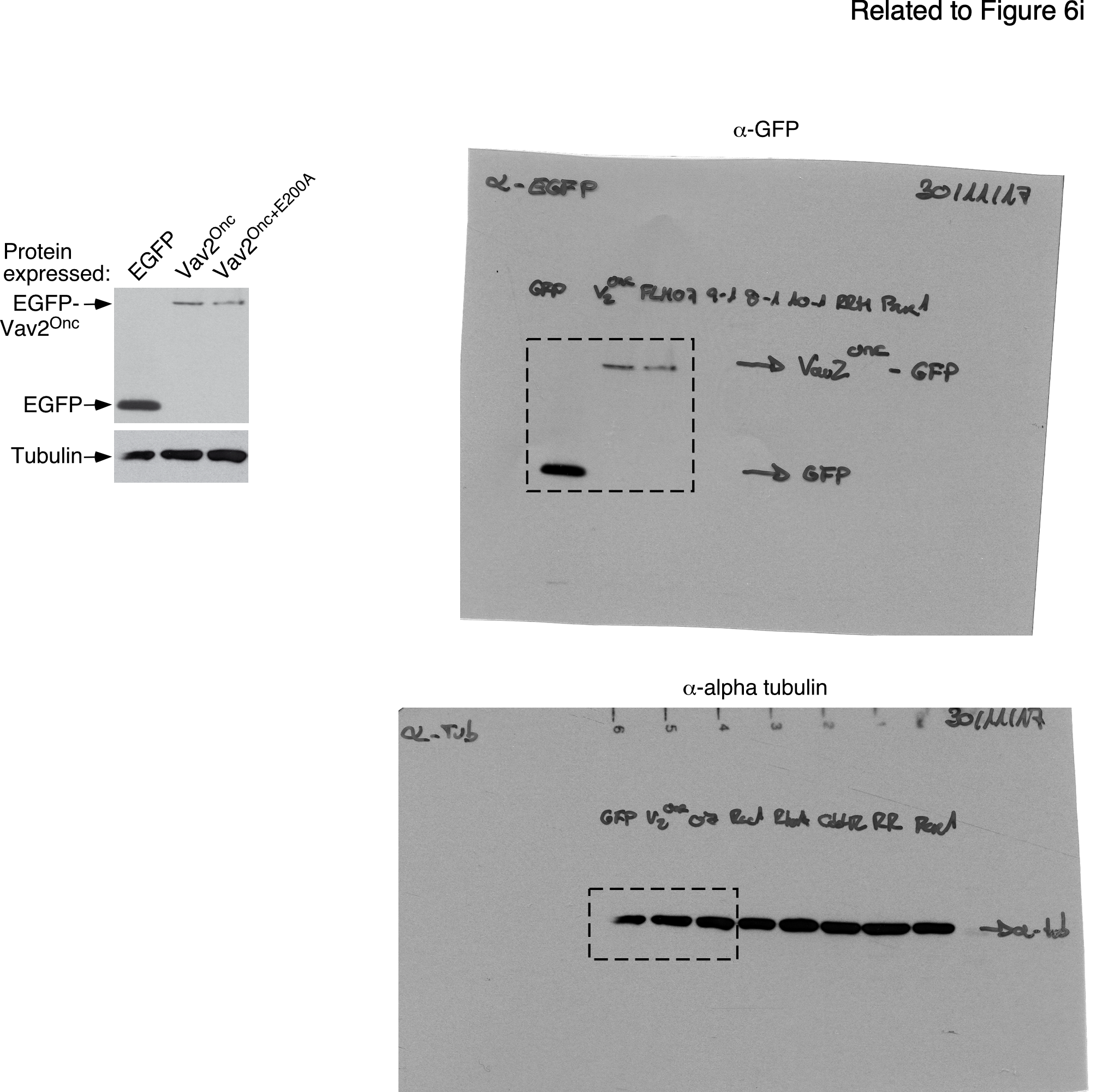

Supplement: Supplementary file 6 — Source Data [file 41467_2020_18524_MOESM6_ESM.zip › 5.4. SOURCE DATA/WB raw data/Figure 6.Raw.WT.tif]
